# Early Life Conditions and Physiological Stress following the Transition to Farming in Central/Southeast Europe: Skeletal Growth Impairment and 6000 Years of Gradual Recovery

**DOI:** 10.1371/journal.pone.0148468

**Published:** 2016-02-04

**Authors:** Alison A. Macintosh, Ron Pinhasi, Jay T. Stock

**Affiliations:** 1 PAVE Research Group, Division of Biological Anthropology, Department of Archaeology & Anthropology, University of Cambridge, Cambridge, United Kingdom; 2 Earth Institute and School of Archaeology, Newman Building, University College Dublin, Dublin, Ireland; Museo Nazionale Preistorico Etnografico 'L. Pigorini', ITALY

## Abstract

Early life conditions play an important role in determining adult body size. In particular, childhood malnutrition and disease can elicit growth delays and affect adult body size if severe or prolonged enough. In the earliest stages of farming, skeletal growth impairment and small adult body size are often documented relative to hunter-gatherer groups, though this pattern is regionally variable. In Central/Southeast Europe, it is unclear how early life stress, growth history, and adult body size were impacted by the introduction of agriculture and ensuing long-term demographic, social, and behavioral change. The current study assesses this impact through the reconstruction and analysis of mean stature, body mass, limb proportion indices, and sexual dimorphism among 407 skeletally mature men and women from foraging and farming populations spanning the Late Mesolithic through Early Medieval periods in Central/Southeast Europe (~7100 calBC to 850 AD). Results document significantly reduced mean stature, body mass, and crural index in Neolithic agriculturalists relative both to Late Mesolithic hunter-gatherer-fishers and to later farming populations. This indication of relative growth impairment in the Neolithic, particularly among women, is supported by existing evidence of high developmental stress, intensive physical activity, and variable access to animal protein in these early agricultural populations. Among subsequent agriculturalists, temporal increases in mean stature, body mass, and crural index were more pronounced among Central European women, driving declines in the magnitude of sexual dimorphism through time. Overall, results suggest that the transition to agriculture in Central/Southeast Europe was challenging for early farming populations, but was followed by gradual amelioration across thousands of years, particularly among Central European women. This sex difference may be indicative, in part, of greater temporal variation in the social status afforded to young girls, in their access to resources during growth, and/or in their health status than was experienced by men.

## Introduction

Human skeletal growth and development are complex biological processes progressing within a genetic/hormonal, environmental, and cultural framework [1–6]. The intimate relationship between human biology and culture is particularly evident in the Holocene, with the development of agriculture as the primary mode of subsistence. Though not a universal pattern, subsistence shifts at the transition to farming have been associated with reduced adult body size and/or skeletal and dental evidence of poor diet and health when compared to the corresponding profiles of related hunter-gatherer populations [7–17]. These studies and others have provided insight into the difficult early life conditions often experienced by the first farming populations as they adjusted to major subsistence, socioeconomic, and demographic change.

There are few inter-population assessments of the trends in adult body size and sexual dimorphism across the transition to farming in Central/Southeast Europe, and Mesolithic remains from Central Europe are scarce. Piontek and Vančata [17] reported a reduction in body size among some Early Neolithic Central European farming groups relative to preceding Mesolithic individuals from Germany, Britain, France, Spain, and Crimea. Similarly, among a larger sample of European Mesolithic and Neolithic populations, Berner and colleagues [18] identified a reduction in sexual dimorphism in stature and body mass across the transition to farming. However, the relationship between changes in body size, genetic, and environmental conditions during early life across the shift to agriculture is not well understood.

In the Iron Gates region, Serbia (Southeast Europe), Mesolithic and Neolithic lifeways coexisted for at least a thousand years, much longer than elsewhere in the Balkans [19]. Here, the Mesolithic period was followed by a Transitional phase (~6200–5900 calBC) during which contact and exchange between Mesolithic and Neolithic populations occurred [20–21]. This period of Neolithic influence, combined with the stable environment and rich and predictable subsistence of the Iron Gates region [19, 22], likely facilitated the development of the relatively large and permanent settlements of Mesolithic foragers found here. In addition to their semi-sedentary lifestyle, Iron Gates Mesolithic communities were also unusual for their advanced architecture, artistic expression, burial customs, and specialized reliance on fishing [23]. Though Late Mesolithic Iron Gates groups are not necessarily representative of hunter-gatherer communities elsewhere in Europe, this transitional Mesolithic-Neolithic semi-settled lifestyle and cultural complexity *prior* to intensive reliance on agriculture and domesticated animals [19, 22, 24] presents an interesting opportunity to explore the impact of food production on early life conditions experienced through the transition to full-scale agriculture in Central and Southeast Europe.

Subsistence in the Late Mesolithic-Transitional phase of the Iron Gates involved the seasonal exploitation of resources and cultivation of wild crops [22], the gathering of floral resources, hunting of red deer, roe deer, wild boar, and elk [24–26] and a heavy reliance on freshwater fish [19, 27–31]. Analyses of growth trajectories among the Late Mesolithic inhabitants of the Iron Gates suggest that early life stress was only moderate, and was short enough in duration to allow for catch-up growth prior to growth plate closure [32]. However, with the movement of intensive agriculture through the Balkans into Central Europe, the diet and health of the human populations here appear to have changed considerably.

The intensification of small-scale cultivation in Central Europe began with the appearance of the Neolithic *Linearbandkeramik* (LBK) cultural group, which emerged in Transdanubia around 5600–5500 calBC [33]. Within 500 years, LBK communities had spread through to France in the west and the Ukraine in the east [25,34–36]. The LBK practiced intensive agriculture and were heavily reliant on C3 plants such as wheat [37–41], supplemented by meat and milk from domesticated animals, particularly cattle, and the continued exploitation of wild game [25, 37, 39, 42–44]. Unlike the Iron Gates Late Mesolithic communities, skeletal and dental evidence from the LBK groups included in this study (Vedrovice, Nitra, Schwetzingen, Stuttgart-Mühlhausen) documents high childhood physiological stress and pathogen load [39, 45–47], particularly among women. These same LBK populations also performed intensive physical activity [48–49] that likely began, at least for some young boys, in early childhood [50]. Thus, existing evidence suggests that early life conditions may have been difficult for these Early Neolithic LBK populations, and it is expected that adult body size and/or patterns of sexual dimorphism will differ from those documented among the Late Mesolithic inhabitants of the Iron Gates. This expectation is supported by the findings of Piontek and Vančata [17]: the German LBK group in their study did exhibit drastically reduced body size relative to Late Upper Paleolithic and Mesolithic European groups as well as to contemporaneous Early Neolithic Corded Ware cultures. Sexual dimorphism in body size was also exceptionally high in this LBK group, reflecting higher values among males.

After this initial period of change with the introduction of farming, cereal agriculture and stock rearing became widespread, providing the main subsistence base for millennia [51–58]. Einkorn, emmer, and bread wheat, hulled six-row barley, millet, spelt, oats, rye, lentils, and peas remained staple cultivars in Central/Southeast European agricultural populations through to the Medieval period [51, 55, 59]. However, the introduction of the plough in the Mid-Late Neolithic increased agricultural productivity, supporting greater population densities and larger communities [43]. The plough also enabled the clearance of larger tracts of land for livestock grazing, and by the Middle Neolithic and Early Bronze Age, archaeological evidence for the intensification of dairying and cheese production becomes visible [52–53]. These demographic and dietary changes drove genetic selection among agricultural populations, particularly in alleles conferring disease resistance [60–61] and enhanced nutrient processing capabilities [62–66]. For instance, evidence of selection for lactase persistence in response to intensive dairying among Central European agriculturalists appears at least by the Late Bronze Age [67], if not slightly earlier [68–69].

Phenotypic variation has also been influenced by dietary change and the development of social complexity after the introduction of farming. Social inequality according to status and sex is often visible in the archaeological record among the farming communities in the current study, regardless of time period, not just in grave goods and burial rites [45, 70–73], but also in health [46, 74–76], and access to plant-based dietary components, milk, milk products, and animal protein [37–41, 77–82]. In particular, differences in the degree to which men and women were afforded social status, and related variation in their overall growth conditions, were common [75, 77, 83–86], and these differences can impact sexual dimorphism in adult body size. Typically, systemic genetic, hormonal, and physiological controls on pubertal growth, skeletal maturation, and final bone length result in the standard levels of body size dimorphism between men and women [87–89]. However, these genetic and hormonal growth trajectories can be impacted substantially by cultural factors, such as differential access to resources by sex [6] or the preferential treatment of offspring, particularly under stressful conditions [90–91]. For instance, at the Iron Age site of Tápiószele in Hungary, and another nearby site, men's stature increased relative to a Bronze Age site from the same region, while women's stature declined [76]. Women also exhibited fewer temporal improvements in health conditions than contemporaneous men and experienced higher childhood morbidity and dental caries than men [76]. Thus, factors such as increasing socioeconomic complexity, population density, and greater reliance on agriculture may have impacted the diet and health of men and women quite differently. Though common among many of the cultures included in the current study, high gender and social inequality was not ubiquitous [92–93]. When present, inequalities in access to resources among the members of the increasingly more socioeconomically complex populations in this study are expected to contribute to trends in body size and/or sexual dimorphism long after the initial shift to farming in Central/Southeast Europe.

Several millennia after the initial onset of farming, better technologies and more efficient methods of crop production, as well as improvements in sanitation and healthcare, all likely increased the availability and reliability of adequate nutrition and reduced the likelihood of infection among agricultural populations [15–16, 94–96]. For example, though sex differences in health and stress were evident at Tápiószele, overall increases in life expectancy at birth and in the first year of life, and reductions in developmental stress among subadults relative to Bronze Age groups, suggest that at least some aspects of health did improve over time [75–76]. Among living humans, similar improvements in diet, health, and socioeconomic opportunity can elicit rapid recovery in growth and adult body size, leading to significant height and body mass differences that can emerge within a single generation following migration from developing to industrialized nations [97–100]. Further, industrialization since the early 19^th^ century has been associated with rapid secular increases in height and body mass with changes in nutrition, hygiene, social class, and stress level [101–112].

It is this impact of early life conditions on adult body size and shape that allows for the interpretation of overall growth history and relative physiological stress from adult skeletal remains. There is undoubtedly an underlying genetic basis to the growth trajectory [1–3], long bone length and stature [3, 5, 113–120], body and limb proportions [4, 91, 121–122], and body mass [3]. However, among living humans, an individual's genetically-determined growth trajectory can be modified substantially by the redirection of energy from growth to more essential functions, eliciting major delays [100, 103–104, 117, 119–120, 123–124]. One of the most powerful sources of growth impairment is the simultaneous effect of childhood malnutrition and infection [125], while the combination of high-impact loading and inadequate energy intake can also have lasting influences on growth and body size [126–130]. The developmental canalization of growth is such that catch-up growth may occur following less severe or prolonged stress, either through a markedly accelerated rate of growth or by the extension of the growth period [2, 103, 119, 131–133]. However, catch-up growth is costly [124, 133] and cannot always make up for previous delays [119, 134]. Thus, proximate and long-term trends in adult body size, limb proportions, and sexual dimorphism among foragers and agriculturalists have the potential to inform on a variety of factors during life, including the combined effects of dietary and health status during growth, relative physiological stress, biological and life history differences between men and women, cultural differences in access to resources, and the sex-specific assignment of tasks.

### Tracking the impact of early life conditions on body size and limb proportions

Archaeological evidence from foraging and agricultural groups in Central/Southeast Europe documents cultural, social, and sex differences in the distribution of grave goods, burial rituals, life expectancy, diet, disease load, dental health, developmental stress, and habitual behaviors [24–26, 48–49, 75–77, 79–82, 84–86, 92–93, 135–139]. These differences suggest that human populations living before and following the introduction of intensive food production in these regions would have experienced variation in the severity of stress experienced during growth, its timing relative to local growth velocities, the available opportunities for catch-up growth prior to adulthood, and ultimately in adult body size/shape. The current study tracks diachronic change and regional differences in mean adult stature, body mass, limb proportions, and sexual dimorphism in these parameters through ~8000 years of subsistence and cultural change in Central/Southeast Europe (~7100 to ~850 AD). Body size and limb proportion variables are interpreted in combination with existing skeletal and dental evidence of developmental stress, health status, access to resources, and habitual behavior among the cultures and cemeteries included. This approach allows for insight to be gained into the impact of agricultural intensification, increasing sedentism, demographic and social change over thousands of years on the early life conditions and overall growth history of young men and women in Central/Southeast Europe.

## Materials and Methods

### Skeletal sample

**Fig 1:**
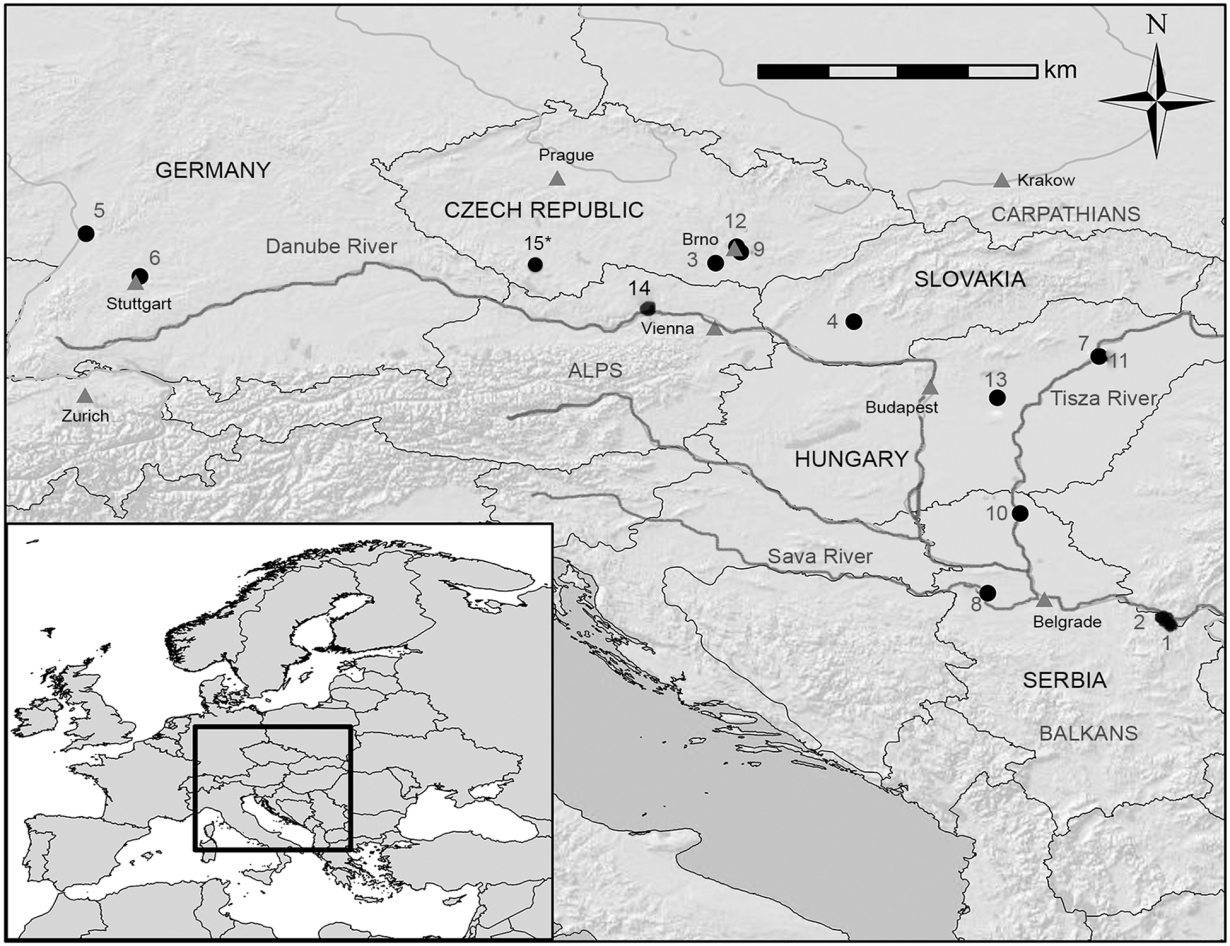
Map of Central/Southeast Europe with geographical location of cemeteries. 1. Vlasac 2. Lepenski Vir 3. Vedrovice 4. Nitra Horné Krškany 5. Schwetzingen 6. Stuttgart-Mühlhausen 7. Polgár-Ferenci-hát 8. Hrtkovci-Gomolava 9. Brno-Tuřany 10. Ostojićevo 11. Polgár Kenderföld 12. Brno-Maloměřice 13. Tápiószele 14. Pottenbrunn 15. Hoštice. *: this site was used only for regional analyses.

The Late Mesolithic-Transitional phase hunter-gatherer-fisher population (~7131–5838 calBC) comprised skeletal remains from the cemeteries of Vlasac and Lepenski Vir in the Iron Gates region. These sites are located three kilometers apart along the Danube River in the Gospodjin Vir gorge in Vojvodina, northern Serbia. Agricultural populations included in this study represent portions of four archaeological time periods following the transition to agriculture: the Early/Middle Neolithic (~5300–4600 calBC), Early/Middle Bronze Age (~2300–1450 BC), Early through Late Iron Age (~850 BC-100 AD), and Early Medieval (~800–850 AD). These cemeteries are primarily located in Central Europe, in modern-day Baden-Württemberg (Germany), Moravia (Czech Republic), Lower Austria, western Slovakia, and the northern Carpathian Basin in Hungary. A further two agricultural cemeteries are located in the southern Carpathian Basin of Southeast Europe, in modern-day Vojvodina (northern Serbia). The Moravian Eneolithic Bell Beaker cemetery of Hoštice 1 za Hanou (~2600–2000 BC) was included in regional analyses for temporal trends within Moravia. The geographical locations of all cemeteries from which the skeletal remains originated are presented in Fig 1.

Details of the cemetery collections utilized in this study are presented in Table 1, and all individuals included in analyses are listed in S1 Table, in which specimen numbers and all relevant individual-level data are also provided. All collections were pre-excavated and are housed in museum or university collections (see Table 1). Please see S1 Appendix for further details on the cemeteries included in analyses. Collections were accessed with permission from the relevant curators. Sex assessment and age estimation was performed according to the methods outlined in Buikstra and Ubelaker [140]. Sex assessments incorporated as many morphological criteria as possible from the cranium and pelvis. In the event of ambiguity, priority was given to pelvic criteria. Neither limb bone dimensions nor any of the variables in which sexual dimorphism was explored were utilized in sex assessment of the skeletons. Individuals sexed as 'Possible Male' or 'Possible Female' were pooled with male and female groups, respectively, for analyses. Only skeletally mature adults with all long bone epiphyses fused were included in this study, with priority given to individuals aged approximately 20–40 years at death.

**Table 1:** Central/Southeast European cemetery details.

Culture	~Date (BC)*	Cemetery	Location	Collection Housed At:	N (male/female)
**Mesolithic**					**52 (25/27)**
*Late-Transitional*					
Lepenski Vir	7131–5838*	Vlasac	Vojvodina, Serbia	University of Belgrade	27 (14/13)
Lepenski Vir	6240–5845*	Lepenski Vir	Vojvodina, Serbia	University of Belgrade	25 (11/14)
**Neolithic**					**141 (87/54)**
*Early*					
LBK	5300–5100*	Vedrovice	Moravia, Czech Republic	Moravian Museum (Brno)	22 (10/12)
LBK	5370–4980*	Nitra Horné Krškany	western Slovakia	Moravian Museum (Brno)	22 (12/10)
ALP	5293–5068*	Polgár-Ferenci-hát	Hungary	Hungarian Natural History Museum (Budapest)	10 (8/2)
LBK	5260–5010* or	Schwetzingen	Baden-Württemberg, Germany	Stuttgart Regional Council, State Conservation Office- Osteology (Konstanz)	30 (16/14)
	5300–5070*				
LBK	5200–4960*	Stuttgart-Mühlhausen	Baden-Württemberg, Germany	University of Tübingen	41 (25/16)
*Middle*					
Vinča	~4950–4600*	Hrtkovci-Gomolava	Vojvodina, Serbia	Museum of Vojvodina (Novi Sad)	16 (16/0)
**Eneolithic**					
Bell Beaker	~2600–2000	Hoštice 1 za Hanou	Moravia, Czech Republic	Masaryk University (Brno)	**12 (9/3)**
**Bronze Age**					**97 (55/42)**
*Early*					
Únětice	2300–1700	Brno-Tuřany	Moravia, Czech Republic	Masaryk University (Brno)	17 (10/7)
Maros	~1600/1500	Ostojićevo	Vojvodina, Serbia	National Museum of Kikinda	56 (28/28)
*Middle*					
Füzesabony	1550–1450	Polgár Kenderföld	Hungary	Hungarian Natural History Museum (Budapest)	24 (17/7)
**Iron Age**					**71 (35/36)**
*Early*					
Bosut	850-600/500	Hrtkovci-Gomolava	Vojvodina, Serbia	Museum of Vojvodina (Novi Sad)	23 (8/15)
*Middle*					
Celtic	400–200	Brno-Maloměřice	Moravia, Czech Republic	Moravian Museum (Brno)	20 (15/5)
*Middle/Late*					
Scythian	385–100 AD*	Tápiószele	Hungary	Hungarian Natural History Museum (Budapest)	28 (12/16)
**Early Medieval**					
Slavic	800–850 AD	Pottenbrunn	Lower Austria	Vienna Natural History Museum	**42 (18/24)**

* indicates calibrated radiocarbon date; *N* = number of individuals; LBK = Linearbandkeramik; ALP = Alföld Linear Pottery; dates from: [20–21,26,36,38,41,75,166,193–204], Zdeněk Tvrdý, pers. comm.

To reduce variation attributable solely to genetic differences in body size and build, latitudinal variation among populations selected for inclusion was minimized, as much of the population specificity of body size and shape variables ultimately stems from long-term adaptation to climatic conditions [4, 141–149]. Further, where possible, temporal sequences were obtained from the same local regions (e.g., Moravia, Vojvodina) or the same cemetery (e.g., Gomolava).

### Bone measurements and estimated body size variables

An osteometric board was used to record maximum lengths of the humerus, radius, femur, and tibia parallel to the long axis of the diaphysis, as well as bicondylar length of the femur, and biomechanical length of the tibia [150–153]. All length measurements were recorded to the nearest 0.5 millimeter. Left and right supero-inferior (S-I) femoral head diameters were measured to the nearest 0.1 millimeter using digital sliding calipers. For Pottenbrunn, some S-I femoral head diameters were obtained directly from three-dimensional laser scans of femora obtained by A. Macintosh using the measuring tool in Rapidform XOR (see Macintosh and colleagues [48] for laser scan methodology).

Variation in the relative lengths of the proximal and distal segments were evaluated using brachial and crural indices, which are the ratio of distal to proximal segment lengths in the upper and lower limb, respectively [148]. Limb proportion indices were calculated using maximum lengths of the humerus, radius, and tibia, and bicondylar length of the femur [145, 147, 154–155). Poor preservation of both left and right elements, most often involving the medial malleoli, required estimation of average maximum element length in eight of 219 brachial indices and 23 of 262 crural indices included in analyses. Individuals for whom approximate average element length was used to calculate limb proportions are identified in S1 Table. Stature and body mass were estimated utilizing the equations for European Holocene populations derived by Ruff and colleagues [153]. These equations were derived for a large sample (stature: N = 501; body mass: N = 1145) spanning the Mesolithic to modern day (~7000 BC to ≥ 1900 AD) across most of Europe, including samples from the Balkans, Czech Republic, Germany, and Eastern Austria. Stature was estimated using maximum femoral length. If the femora were missing or too poorly preserved to provide a length measurement, tibial maximum length was used. However, ecogeographic variation in distal limb lengths means that region-specific equations are required when estimating stature from the tibia [153]. In the current study, the 'northern' equation of Ruff and colleagues [143] was used to estimate stature in 7% of German, Czech, Slovakian, and Austrian individuals (14 of 187 individuals), and the 'southern' equation was used in 5% of Serbian and Hungarian individuals (10 of 193 individuals). These individuals are indicated in S1 Table.

Body mass was estimated from femoral head SI breadth utilizing the equations for European Holocene populations derived by Ruff and colleagues [153]. Among Medieval individuals for whom S-I breadths were measured directly from 3D laser scans, measurements were taken on two separate occasions one year apart (AAM) and the average value was used to estimate body mass. Body mass estimates quantified from both scan- and caliper-derived S-I breadths did not differ significantly in a test sample of femora for which both measurements were available, so all Medieval body mass estimates were pooled together for analyses. Individuals for whom body mass was estimated from a laser scan model are indicated in S1 Table. No age adjustment factor was applied to stature or body mass estimates, following Ruff and colleagues [153]. Where possible, all estimated body size variables were calculated from an average of left and right side elements, in order to reduce laterality biases and minimize the effects of asymmetry [152–153, 155–156].

### Sexual dimorphism

Mean stature and body mass were expected to be larger in men than women simply due to genetic and hormonal differences between the sexes [87, 89, 157–158]. Thus, the current study investigated change in a ratio of male to female size that expresses the male mean as a percentage of the female mean for a given variable: (male mean / female mean)*100 [159–160]. However, this index does not take sample size or standard deviation into account. Thus, in addition, a measure of sexual dimorphism quantified using the regions of non-overlap of male and female distributions was employed (D) [161], whereby values range from 0 (no dimorphism, complete overlap) to 1 (completely dimorphic, no overlap). Further, effect sizes for sexual dimorphism were calculated using Pearson's correlation coefficient *r*, according to the following method outlined by Mascie-Taylor [162]:

$$r=\sqrt{\frac{t^{2}}{t^{2}+df}}$$

where df = *n*-2 and

$$t=\frac{{\bar{X}}_{m}-{\bar{X}}_{f}}{\sqrt{\frac{{\left(SD_{m}\right)}^{2}}{n_{m}}\;+\;\frac{{\left(SD_{f}\right)}^{2}}{n_{f}}}}$$

An *r*-value greater than 0.8 was considered a large effect size, indicating a high magnitude of difference between males and females, and a value of 0.5 was considered a medium effect [162]. These effect sizes are more conservative than the widely followed suggestions of Cohen [163–164], whereby any *r*-value greater than 0.5 is considered a large effect, so all values >0.5 are highlighted in the results tables.

### Analytical methods

Sample data distributions were assessed for normality using z-scores for skewness and kurtosis, Q-Q plots, and histograms. All outliers more than three standard deviations from the mean were removed and all data included in analyses were normally distributed. One-way analysis of variance (ANOVA) was used to test for temporal change in body size variables and limb proportions among male and female Central/Southeast Europeans. Post-hoc comparisons were performed with either the Hochberg's GT2 or Games-Howell test. Independent samples *t*-tests were used to explore sexual dimorphism in body size variables and limb proportions. Statistical analyses were conducted in SPSS v23, and statistical significance was considered as a *p*-value less than 0.05.

## Results

### Trends by time period

**Fig 2:**
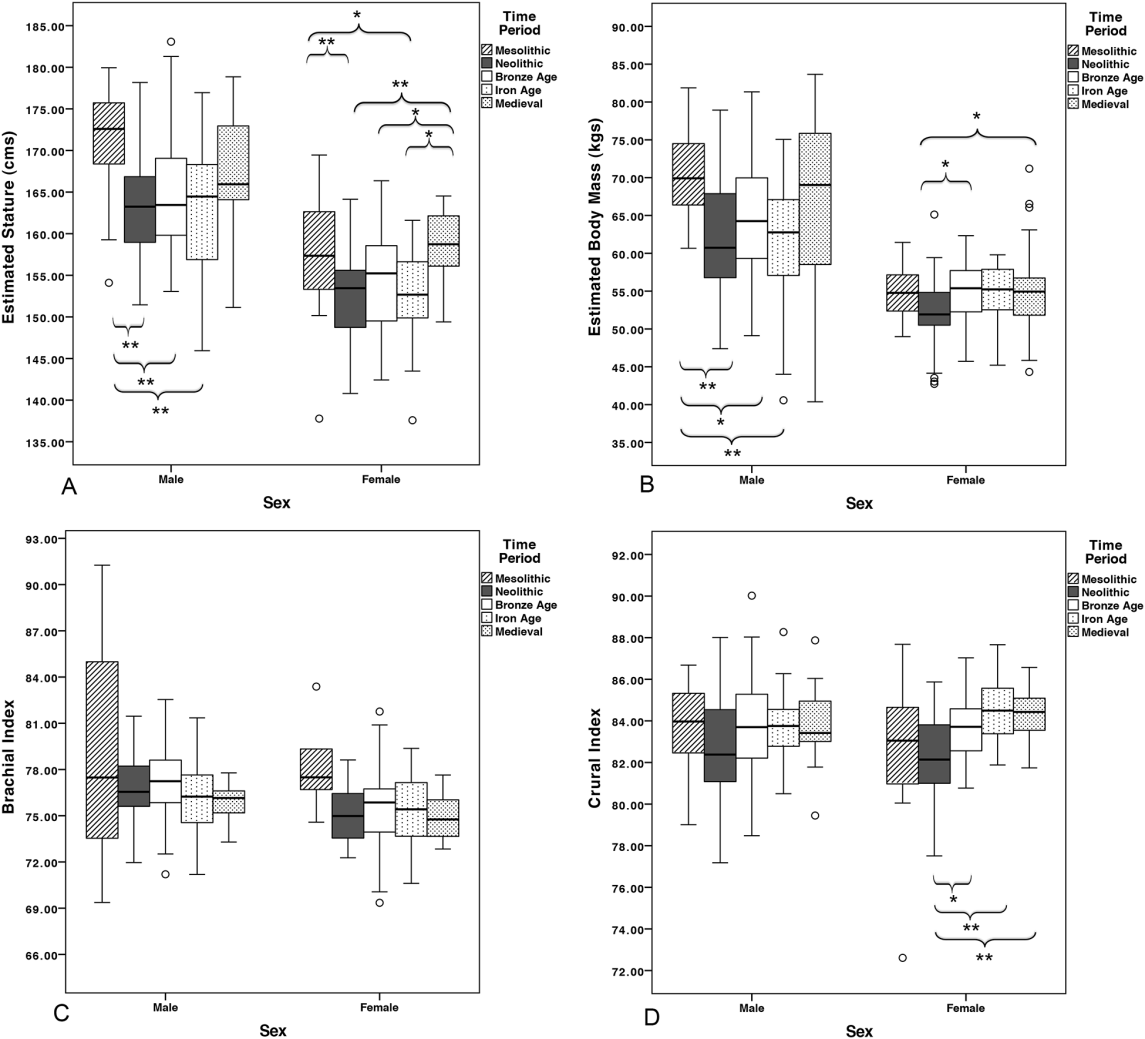
Estimated body size variables by time period and sex. A) Stature, B) Body mass, C) Brachial index, D) Crural index. Brackets indicate significant differences (* = *p*<0.05; ** = *p*<0.01).

Summary statistics for body size and limb proportion variables by sex and time period are presented in Table 2 and results of one-way ANOVAs by sex and time period are presented in Table 3. Boxplots for temporal change by sex and time period in stature, body mass, brachial index, and crural index are presented in Fig 2. When agricultural populations are compared to the Late Mesolithic hunting/gathering/fishing group, the relatively large body size of the latter is apparent, particularly among men. Late Mesolithic Iron Gates men are significantly taller (*p*<0.001) and heavier (*p*<0.006) than Neolithic men, significantly taller (*p*<0.001) than Bronze Age men, and significantly taller (*p*<0.018) and heavier (*p*<0.021) than Iron Age men. Similarly, Late Mesolithic women are significantly taller than both Early Neolithic (*p*<0.001) and Iron Age (*p*<0.013) women. Though mean brachial and crural indices do not change significantly, these mean values are also higher in Late Mesolithic men and women than their Neolithic counterparts.

**Table 2:** Summary statistics for body size variables by sex and time period.

		Stature (cms)			Body Mass (kgs)			Brachial Index			Crural Index	
	N	Mean	SD	N	Mean	SD	N	Mean	SD	N	Mean	SD
**MALES**												
Mesolithic	23	171.15	6.82	16	70.47	6.12	8	79.08	7.46	7	83.60	2.70
Neolithic	76	163.36	5.65	81	62.11	7.52	57	76.77	1.98	62	82.68	2.23
Bronze Age	54	164.58	6.83	50	64.38	7.70	37	77.16	2.49	42	83.91	2.31
Iron Age	26	163.46	7.17	32	61.75	7.86	8	76.18	2.98	18	83.87	1.82
Medieval	17	166.61	7.23	17	66.38	11.9	15	75.89	1.12	17	83.72	1.90
**FEMALES**												
Mesolithic	27	157.46	6.75	10	55.08	3.56	5	78.30	3.31	9	82.42	4.48
Neolithic	50	152.00	5.50	50	51.94	4.46	32	74.92	1.95	34	82.24	2.04
Bronze Age	40	154.36	5.69	38	54.96	4.35	29	75.58	2.72	30	83.71	1.54
Iron Age	32	152.77	5.39	35	54.52	4.00	16	75.26	2.54	18	84.54	1.52
Medieval	23	158.52	4.31	21	55.60	6.99	11	75.01	1.68	21	84.32	1.29

N: number of individuals; SD: standard deviation.

**Table 3:** Results of one-way ANOVAs by sex and time period.

		Pair-wise comparisons								
		Meso vs.			Neo vs.			BA vs.		IA
	ANOVA	Neo	BA	IA	BA	IA	Med	IA	Med	Med
**Males**										
Stature	<0.001	<0.001	<0.001	<0.001	ns	ns	ns	ns	ns	ns
Body Mass	<0.001	<0.001	<0.22	<0.001	ns	ns	ns	ns	ns	ns
**Females**										
Stature	<0.001	<0.001	ns	<0.016	ns	ns	<0.001	ns	<0.05	<0.02
Body Mass	<0.012	ns	ns	ns	<0.048	ns	<0.044	ns	ns	ns
Crural Index	<0.001	ns	ns	ns	<0.016	<0.001	<0.001	ns	ns	ns

values represent the *p*-values of ANOVAs or post-hoc tests significant at an α of 0.05; ns = not significant; Meso: Mesolithic; Neo: Neolithic; BA: Bronze Age; IA: Iron Age; Med: Medieval.

Through the ~6150 years spanned by the agricultural populations examined in this study (Early Neolithic through Early Medieval periods), average male body size does not change significantly. However, Neolithic men consistently have the lowest mean estimated stature and body mass of all agriculturalists. Among Neolithic women, deficits in body size variables and crural index are particularly pronounced relative to subsequent agricultural women. Neolithic women have significantly lower mean estimated stature than Early Medieval women (*p*<0.001), significantly lower mean body mass than Bronze Age women (*p*<0.012), and significantly lower mean crural indices (shorter tibiae relative to femora) than Bronze Age (*p*<0.012) and Early Medieval (*p*<0.001) women. Early Medieval women are also significantly taller on average than their counterparts in the Iron Age (*p*<0.007).

Results of independent samples *t*-tests for sexual dimorphism in body size and limb proportions by time period are presented in Table 4. Males at all time periods examined have significantly higher stature and body mass than females (*p*<0.001 for all). Effect sizes for the magnitude of sexual dimorphism in stature and body mass are very large in the Mesolithic period (*r*-values of 0.72 and 0.86, respectively), and decline consistently through to the Early Medieval period (*r*-values of 0.55 and 0.42, respectively). Temporal declines in D values for stature and body mass also indicate that male and female distributions for these variables overlap to a progressively greater extent through time, becoming more similar. Indices of sexual dimorphism parallel this trend to a certain extent, declining consistently from the Mesolithic through Bronze Age for both variables. By contrast, significant sexual dimorphism in limb proportions is rare, though brachial index is significantly higher among Neolithic (*p*<0.001) and Bronze Age (*p*<0.017) males than females. Effect sizes are also small (most *r*<0.2, maximum of 0.4) in almost all instances for limb proportions, as are D values, and neither indicate any consistent trend through time in the sexual dimorphism of limb proportions.

**Table 4:** Sexual dimorphism in body size variables by time period.

	Stature				Body Mass			
	*p*	Index	*r*	D	*p*	Index	*r*	D
**MESOLITHIC**	<0.001	108.69	*0*.*716*	0.687	<0.001	127.94	***0*.*856***	0.888
**NEOLITHIC**	<0.001	107.47	*0*.*710*	0.692	<0.001	119.58	*0*.*650*	0.604
**BRONZE AGE**	<0.001	106.62	*0*.*636*	0.586	<0.001	117.14	*0*.*616*	0.566
**IRON AGE**	<0.001	106.99	*0*.*644*	0.605	<0.001	113.26	0.493	0.458
**MEDIEVAL**	<0.001	105.10	*0*.*554*	0.517	<0.001	119.39	0.423	0.432
	Brachial Index				Crural Index			
	*p*	Index	*r*	D	*p*	Index	*r*	D
**MESOLITHIC**	ns	101.00	0.078	0.058	ns	101.43	0.172	0.131
**NEOLITHIC**	<0.001	102.47	0.418	0.362	ns	100.54	0.100	0.082
**BRONZE AGE**	<0.017	102.09	0.291	0.238	ns	100.24	0.053	0.041
**IRON AGE**	ns	101.22	0.157	0.132	ns	99.21	0.201	0.044
**MEDIEVAL**	ns	101.17	0.294	0.247	ns	99.29	0.183	0.149

*p*: *p*-value of *t*-test; Index: Index of sexual dimorphism; *r*: correlation coefficient of effect size: large effect = ≥0.8, medium effect = 0.5, small effect = 0.2; large effect sizes are bolded; medium to large effect sizes are in italics; D: measure of overlap between male and female distributions, ranging from 0 (complete overlap, no dimorphism) to 1.0 (no overlap, complete dimorphism).

### Trends by region

Summary statistics for body size and limb proportion variables are presented for men and women by geographic region and cemetery in S2 Table. Results for regional differences within each time period by sex are presented in Table 5. Among Iron Gates foragers, women at Lepenski Vir are of significantly lower stature (*p*<0.001) and body mass (*p*<0.011) than women at Vlasac, while men have significantly reduced crural indices (*p*<0.032). Among Neolithic farming populations, there are almost no significant differences in body size or limb proportions among the LBK, ALP, and Vinča men and women, despite these groups spanning Germany to Vojvodina. Hungarian ALP men at Polgár-Ferenci-hát have significantly reduced brachial indices compared to LBK men in Moravia (Vedrovice; *p*<0.034) and Baden-Württemberg (Schwetzingen; *p*<0.033), but this difference is not replicated in any other body size or proportion variable. In the Bronze and Iron Ages however, Central and Southeast Europe diverge; in these time periods, men and women from Vojvodina exhibit significantly smaller body size and/or relatively shortened distal lower limb segments relative to broadly contemporaneous groups in Central Europe (Moravia and Hungary; see Table 5). There are never any significant differences between Hungarian and Moravian groups for any variable in either sex. Within Moravia/Lower Austria, the Eneolithic population from Hoštice does not differ significantly in body size or limb proportions from preceding or subsequent agriculturalists in the region. However, regional analyses must be interpreted with caution, as the division of time periods into constituent cemeteries greatly reduces sample size, particularly among the female group and at certain cemeteries. Sampling biases may be affecting results among these small cemetery samples, such as at Polgár-Ferenci-hát, Hoštice, and Gomolava.

**Table 5:** Significant results of one-way ANOVAs examining regional differences within time periods by sex.

	ANOVA		
Variable	Males	Females	Pairwise Comparisons
*Mesolithic*			
Stature*	ns	<0.001	**LEP** < **VLA**
Body mass*	ns	<0.011	**LEP** < **VLA**
Crural index*	<0.032	ns	**LEP** < **VLA**
*Neolithic*			
Brachial index	<0.008	ns	**PFH** < **VED** 0.034; **PFH** < **SCH** 0.033
*Bronze Age*			
Stature	ns	<0.001	**OST** < **BT** 0.001; **OST** < **PK** 0.003
Crural index	<0.002	ns	**OST** < **BT** 0.004; **OST** < **PK** 0.031
*Iron Age*			
Stature	<0.001	ns	**GOM** < **BM** 0.001; **GOM** < **TAP** 0.001
Body mass	<0.015	<0.004	Males: **GOM** < **TAP** 0.014; Females: **GOM** < **TAP** 0.006

*: independent samples *t*-tests were used; VLA: Vlasac; LEP: Lepenski Vir; PFH: Polgár-Ferenci-hát; VED: Vedrovice; SCH: Schwetzingen; OST: Ostojićevo; BT: Brno-Tuřany; PK: Polgár Kenderföld; GOM: Gomolava; BM: Brno-Maloměřice; TAP: Tápiószele; values indicate *p-*values of ANOVAs and pairwise comparisons.

## Discussion

This study explores the impact of the transition to farming and ~6150 years of agricultural intensification and cultural change on adult body size and limb proportions among men and women in Central/Southeast Europe. Mean stature and body mass were significantly lower among Neolithic agriculturalists than Late Mesolithic hunter-gatherer-fishers, with particularly large body size among Late Mesolithic men and particularly small body size among Neolithic women. Following the Neolithic period, mean stature, body mass, and crural index among agricultural populations increased very gradually across thousands of years in both sexes, but significantly so almost exclusively among women. Though men at all time periods were significantly taller and heavier than women, the extent to which this was the case was highest among Late Mesolithic foragers and Neolithic farmers, and declined through time among agriculturalists alongside temporal increases in female body size.

Interestingly, among women, mean stature and body mass was significantly higher at Vlasac than Lepenski Vir in the current study, as were male crural indices, despite the contemporaneity of the sites and their close proximity to each other. Existing reconstructions of stature among men and women at Vlasac using the equations of Trotter and Gleser [165] provided mean statures slightly taller than those in the current study: 176.6 cms for males and 163.2 cms for females [28]. These are significantly taller than stature estimates from Western European Mesolithic populations, and would not be expected in a population experiencing high nutritional stress and/or lacking opportunities for catch-up growth [28]. At Vlasac, most human remains have thus far dated to the Late Mesolithic, with its most intensive occupation occurring from ~7131 to 5838 calBC, while Neolithic Starčevo pottery does not appear at Vlasac until after ~6000–5800 calBC [26, 166]. In contrast, human occupation at Lepenski Vir was most intensive during the Transitional phase (Lepenski Vir I-II), between ~6240–5845 calBC [166]. The appearance of the 'Neolithic package' at the site dates to only slightly later: the remains of domesticated pigs, goats, and cattle found in association with Early Neolithic features, such as domed ovens, at Lepenski Vir date to ~6005 to 5798 calBC [166]. The Transitional phase of habitation at Lepenski Vir also contains both Early Neolithic pottery and polished stone axes typical of Neolithic groups [20, 167–168]. This clear documentation of contact between Mesolithic and Neolithic cultures at Lepenski Vir suggests the possibility that hunting/fishing populations living at the site may have experienced some of the same factors that contributed to reduced body size and limb proportions among Early Neolithic groups.

Individuals with non-local trace element signatures have also been identified during the Transitional phase at Lepenski Vir [169], indicative of individuals being buried at the site that originated outside of the Iron Gates. Also, the Iron Gates at this time experienced wetter conditions and considerable flooding [29, 170], likely contributing to evidence for the exploitation of a wider array of dietary resources among post-contact individuals at Lepenski Vir [28, 30–31, 171], with a significantly higher proportion of terrestrial foods in the diet at this time [28]. In addition to any of the above factors, results could also be reflecting the small sample sizes created by the division of time periods into constituent cemeteries. There are also a range of biases known to typify archaeological assemblages, including differential preservation of human remains from different age groups, partial samples of a larger cemetery, variations in the demographic profiles of the cemetery samples, and the overall selectivity affecting the formation of any mortuary sample [172]. It is possible that such biases, which cannot be assessed at the present study, contributed to some extant to the observed temporal patterns, both temporally and regionally. However, none of the above-mentioned factors varies in its direction and intensity between the time periods analysed here.

Despite differences in body size and crural indices between Vlasac and Lepenski Vir, the majority of the Mesolithic group overall was typified by large body size and high limb proportion indices relative to most of the agricultural populations analyzed. The semi-settled and socially complex lifestyle of the Late Mesolithic Iron Gates foragers is not representative of typical European hunter-gatherer lifeways, and their body size appears to be larger than their Western European Mesolithic counterparts [27, 173]. However, the reduction in mean stature and body mass between Late Mesolithic Iron Gates foragers (Southeast Europe) and Neolithic Central/Southeast European agriculturalists (predominantly LBK) identified in this study was similar to that identified by Piontek and Vančata between Mesolithic European groups and Early Neolithic Central European LBK [17]. Further, the large changes in body size identified in the current study between Late Mesolithic and Neolithic populations do parallel existing evidence of substantially different early life conditions for these populations. For instance, the moderate early life stress and/or opportunities for catch-up growth in the Late Mesolithic Iron Gates foragers [32] contrast sharply with high childhood developmental stress, shifting of the childhood growth spurt, and high pathogen load documented among the Early Neolithic LBK at Vedrovice and Stuttgart-Mühlhausen [45–47]. The Early Neolithic LBK populations in this study also show evidence of high lower limb bone loading among both men and women relative to the subsequent agricultural groups, likely associated with higher terrestrial mobility [48]. There is also evidence that high mobility was a component of the early childhoods of at least some young LBK boys [50]. This intensive physical activity, undertaken at a young age in at least some individuals, may have affected already limited energetic resources during growth among LBK populations. It is probable that the combination of a homogeneous carbohydrate-rich diet, high developmental stress, intensive physical activity at a young age, and sex differences within these all contributed to the impairment of adult body size and crural index among Neolithic populations relative to both hunter-gatherers and subsequent farming populations.

Despite large body size in most Iron Gates men and women, particularly at Vlasac, compared to their agricultural counterparts, results suggest that disparity in growth conditions between the sexes may have been present that negatively affected women. The magnitude of sexual dimorphism in stature and body mass in the Iron Gates Late Mesolithic group was the largest of all time periods examined: men were, on average, 128% the body mass of women, a very large effect (*r* = 0.86). Significant differences have been documented in male and female diet at Vlasac and Lepenski Vir [28] that may be playing a role in this result. Similar to the trends documented by Berner and colleagues [18], the overall magnitude of sexual dimorphism in stature and body mass in Central/Southeast Europe decreased slightly across the transition to farming. However, effect sizes for dimorphism in stature and body mass among Neolithic communities overall was still high (*r*>0.6 for body mass; *r*>0.7 for stature), and this was the case across both the LBK and ALP and across a geographic region spanning from southwest Germany to Hungary (e.g., r = 0.94 for stature at Schwetzingen, Nitra, and Polgár-Ferenci-hát).

The substantially smaller body size of Early Neolithic agricultural women relative to men parallels evidence for sex differences in diet and health among these populations, with negative effects for women. Relative to men, LBK women consumed less animal protein, had a higher frequency and severity of dental caries, and had a reduced life expectancy [36–41, 45–46, 74, 78]. Thus, despite the different subsistence systems of Mesolithic and Neolithic populations in the current study, gender inequality that negatively affecting the growth and development of young girls may have been a shared factor of both of these early and socially complex ways of life. Sex differences in the adequacy of resources supplied to young boys and girls, in levels of early life stress, health status, divisions of labor, age at initiation of work, and/or life history could all be playing a role in this particularly high sexual dimorphism in adult body size. Sex differences in sensitivity to poor conditions during childhood and in the likelihood of subsequent recovery could also be playing a role, though the relative impairment of female body size does not appear to support the typical suggestion of higher sensitivity among males [90, 105, 107–108, 123, 134, 174–175].

Following the initial introduction of agriculture, the magnitude of sexual dimorphism declined progressively through time, alongside gradual increases in mean stature, body mass, and crural index that were significant almost exclusively among women. From the Neolithic to the Bronze Age, women's mean body mass increased significantly, and their tibiae became significantly longer relative to femora, a trend which continued into the Iron Age as well. These temporal increases in mean body size/proportions were consistent across Moravia/Lower Austria, inclusive of the Eneolithic sample, and the Carpathian Basin of Hungary and western Slovakia, but were less pronounced within Vojvodina, where Bronze and Iron Age men and women were of relatively small body size. The regional similarity of Neolithic groups throughout Central and Southeast Europe may suggest that the adoption of farming initially had widespread negative impacts on growth and adult body size and shape. Subsequent regional variation in the Bronze Age corresponds to a time of greater socioeconomic complexity and technological innovation [43, 58, 176–181], changing behaviors and divisions of labor for women [76, 139, 182–183] and major migration and admixture events [67, 184–187], that all likely played a role in driving temporal and regional change in body size variables.

The large average body size and high limb proportions of Bronze Age women in Moravia and Hungary, and the low magnitude of sexual dimorphism at this time, do correspond to existing evidence from these regions of relatively high gender equality [183], similar dental health/diet between the sexes, and low physiological stress during growth at this time [76]. For example, by the Bronze Age, the small Polgár region of Hungary was at the intersection of important east-west and north-south trade links, capable of sustaining several Füzesabony communities and their richly furnished cemeteries within just a few kilometers of each other [182]. Socioeconomic conditions were likely good for the inhabitants of these communities, including at Polgár Kenderföld, and women here could clearly attain high social status; the richest grave at the site was that of a 35–40 year old woman, adorned with amber and bronze artifacts [182]. Good conditions in the Polgár region during the Bronze Age, particularly for women, correspond to an increase of ~ nine cms in average female stature across the ~3500 years separating the Füzesabony women here from their ALP counterparts in the Early Neolithic.

Recent genetic evidence has also begun to shed light on major population migrations and admixture between the Neolithic/Eneolithic and Bronze Age periods in Europe, mainly from the Eurasian Steppe regions such as Western Russia and Ukraine [67, 184–187]. An influx of genetically different populations could clearly be contributing to divergence in the body size of Central and Southeast European populations in the Bronze Age. In addition, the gateway position of Vojvodina between influences from both central and southeastern Europe encouraged cultural mixing [188–189], and the genetic diversity of its inhabitants may have been impacted in different ways than the inhabitants of Central Europe.

The Iron Age also brought an influx of eastern cultural influences and genes into the Carpathian Basin [67, 188], coinciding with the movement of Scythian groups from the Eurasian steppes [190], and Celtic migration was also high [188, 191] The small mean body size of Iron Age Bosut men and women from Gomolava relative to their Scythian and Celtic counterparts in Central Europe is thus notable here. In particular, mean stature among Bosut men from Gomolava was very low, so much so that they differed little from Bosut women, and sexual dimorphism in stature at the site was consequently minimal (r = 0.11). There were no significant differences in any body size variables between 'Males' and 'Possible Males' at the site that would suggest the erroneous presence of females in the male sample. However, despite the unusually low body size of the Bosut men in this study, mean male crural index rose significantly in the ~3750 years separating the Neolithic Vinča and Iron Age Bosut men at Gomolava (*p*<0.047). Because childhood malnutrition impacts the growth of the distal limb segments in particular [107], gradual increases in mean crural index among agricultural men in Vojvodina through time could be indicative of declining early life stress. It is likely that sampling biases are affecting the male sample of Bosut from Gomolava, and more research is needed to characterize body size among this Early Iron Age population in Vojvodina, as few Bosut skeletal remains are known from this time in the region [192].

Despite the high degree of migration among Scythians and Celts, the Iron Age population overall did not differ significantly in body size from that of any other agricultural group, though crural indices continued to increase relative to the Neolithic among women. Among the Celts and Scythians, it is rather the differential treatment of men and women that is interesting, as the Iron Age group overall exhibited a relatively high degree of sexual dimorphism in estimated stature. Warrior status was an important social differentiator among Celtic men at the necropolis of Brno-Maloměřice [93], and Celtic warriors were afforded better access to animal protein than non-warriors [81–82]. High social status was not restricted to men, but it does appear to have taken more time for women to attain [92–93]. It is unknown if any of the Celtic men included in analyses from Brno-Maloměřice were warriors, but the greater likelihood of attaining high social status among young men than women at this time could be contributing to the high sexual dimorphism in stature at Brno-Maloměřice (*r* = 0.75) relative to the other Bronze Age, Iron Age, and Medieval sites examined (*r* values ranging from 0.11 to 0.73), with the exception of Tápiószele. At the Iron Age Scythian site of Tápiószele in Hungary, conditions for women relative to men were very poor, and the effect size for stature at the site was correspondingly very large (r = 0.84). As described above, Scythian women here showed evidence of greater childhood morbidity, an earlier average age of developmental stress, and a higher reliance on carbohydrates than men [75], and experienced fewer temporal improvements than did men in body size, levels of developmental stress and dental caries, and health status relative to a Bronze Age sample from the same region [76]. These poor conditions for women relative to men could be contributing, at least in part, to the high levels of sexual dimorphism in body size at Tápiószele.

Though there is still evidence of high dietary and environmental stress during growth at the Early Medieval Slavic site of Pottenbrunn [84, 135], women here were significantly taller than those of the preceding Iron Age group overall. Among men, it was also the Medieval period in which was found the highest mean stature and body mass of all agricultural time periods examined. This large body size, particularly among women, could again reflect genetic differences, as the Slavs were an immigrant population into eastern Austria, arriving sometime around the late 6th or 7th centuries AD [84]. However, in stark contrast to the earliest farming populations in this study, women at Pottenbrunn exhibited *fewer* indicators of developmental stress than men, and there is no isotopic indication of any dietary differences between the sexes at the site [84]. Correspondingly, effect sizes of sexual dimorphism in the Medieval group were the lowest of all time periods, and mean estimated stature among women at Pottenbrunn was second only to pre-farming Late Mesolithic foraging women from Vlasac. In fact, the average Medieval individual at Pottenbrunn could expect to live a decade longer than the average individual at the Early Neolithic cemetery of Vedrovice from ~6150 years earlier (average life expectancy of 37.7 years relative to 27.6 years, respectively) [46, 84]. Thus, developmental insults at Pottenbrunn may have been less severe than those experienced by earlier agricultural groups, or recovery may have been possible prior to growth plate closure, reducing the impact of these insults on adult body size.

However, the relationship between human growth and development and culturally driven environmental change since the transition to agriculture is complex, and it is interesting to note the gradual nature with which body size and proportions changed across the ~8000 years examined by this study. For instance, following impairment in the earliest stages of farming, average body size and limb proportions among men changed very little across thousands of years, despite advances in agricultural efficiency, technology, and sanitation. Even the more pronounced positive temporal change among agricultural women was very gradual, and young girls in even some of the more recent agricultural populations examined (e.g., Tápiószele) still appeared to have had profoundly more severe or prolonged episodes of early life stress than contemporaneous men. However, ultimately, following a pronounced reduction in body size among early farmers relative to foragers, gradual temporal increases in mean stature, body mass, and crural index and reductions in sexual dimorphism among most Central/Southeast Europe agriculturalists do suggest that the overall impact of millennia of cultural, socioeconomic, and technological change was positive, particularly for women.

## Conclusion

This study examined both overall and regional trends in adult body size, limb proportions, and sexual dimorphism among Central/Southeast European foragers and farmers in the context of existing evidence of diet and health from the same populations. This study documented significant reductions in mean stature and body mass among the earliest Neolithic agriculturalists in Central/Southeast Europe relative to preceding Late Mesolithic Iron Gates hunter-gatherer-fishers. The relatively large body size of Late Mesolithic Iron Gates hunter-gatherer-fishers, particularly at Vlasac, contrasted with the relatively small body size and low crural indices of Neolithic agricultural men and women. These deficits in body size and limb proportions among the earliest farmers were followed by gradual increases in mean values across thousands of years, though these were significant almost exclusively among women and were not documented in the Bronze and Iron Ages of Southeast Europe. Particularly high magnitudes of sexual dimorphism in mean stature and body mass in the Late Mesolithic and Neolithic periods suggested that young women at these times were more likely than young men to experience more severe and/or prolonged developmental stress and poor health. These deficits among women relative to men declined through time alongside significant increases in the body size of agricultural women. The very gradual nature by which body size and limb proportions changed through time among agriculturalists, and the regional differences identified in the Bronze and Iron Ages, suggests that temporal improvements to technology and agricultural efficiency, as well as to factors like infrastructure and sanitation, did not necessarily equate to universal, rapid, or population-wide increases in body size. This may reflect the influence of increasing socioeconomic complexity and urbanization on the unequal distribution of resources among individuals and on disease load, and of major admixture and migration events in the Bronze and Iron Ages. However, by the Early Medieval period, mean body size and crural index among both men and women overall had increased markedly from the introduction of farming in Central/Southeast Europe, significantly so among the women. These results parallel dramatic improvements in average life expectancy already documented across the ~6150 years of agriculture covered by this study, and track alongside existing evidence for improved health and access to dietary resources through time among at least some members of Central/Southeast European farming communities.

## Supporting Information

S1 Appendix Supplementary Information on Cemeteries. (DOCX)

S1 Table Body size and limb proportion variables by cemetery and individual. (XLSX)

S2 Table Regional summary statistics for body size variables by sex and site. (DOCX)
