# Supplementary material for: Early Life Conditions and Physiological Stress following the Transition to Farming in Central/Southeast Europe: Skeletal Growth Impairment and 6000 Years of Gradual Recovery
Source: PLoS One. 2016 Feb 4;11(2):e0148468. doi: 10.1371/journal.pone.0148468 (PMC4742066; doi:10.1371/journal.pone.0148468)
Supplement: S1 Appendix — (DOCX) [file pone.0148468.s001.docx]

**Late Mesolithic - Transitional phase**

*Vlasac (Iron Gates, Serbia)*

Vlasac was first discovered in 1970, on a terrace in the Gospodjin Vir Gorge in the Iron Gates region of present-day northern Serbia [1]. The first two seasons of excavation were performed in 1970-1971, revealing 87 graves with 119 individuals, 43 dwelling structures, and 35,000+ objects [1]. Further excavation at the site was performed between 2006 and 2009 [2]. The most recent AMS dating, adjusted for reservoir effect, comes from features found during this most recent period of excavation, and dates the most intensive period of occupation at Vlasac to ~7131-6823 cal BC to 6006-5838 cal BC [2]. This corresponds to the Late Mesolithic (~7300- 6200 cal BC) and Transitional phase (~6200-5900 cal BC or perhaps even shorter, ~6140-5980 cal BC) occupation of the site [1-5].

Raw materials from Vlasac, and neighboring Lepenski Vir (see below) included flint, quartz, obsidian, stone, bone, and antler, from which were constructed a wide variety of tools used for hunting/fishing, in dwelling and settlement uses, everyday life and outdoor activities, and the manufacture of other implements [1]. The dwellings and graves at Vlasac also contained ~100 sacred objects of specific design and elaborate painting/engraving and the remains of many domesticated dogs. Settlement and dwelling structures at Vlasac include surface dwelling structures with floors of crushed limestone and sand, sunken/semi-subterranean dwellings with simple hearths and hearth structures, remains of tents, and stone structures in open areas (possibly working areas) [1]. Burials at Vlasac were varied, most typically single and multiple inhumations, but also cremations and secondary burials of men and children [1].

**Late Mesolithic - Transitional phase**

*Lepenski Vir (Iron Gates, Serbia)*

The site of Lepenski Vir was located three kilometers downstream from Vlasac, and was discovered in 1960 on a sloping terrace on the right bank of the Danube River [6-7]. Excavation of the site began in 1965, led by Dragoslav Srejović. A total of ~2500 m^2^ has been excavated, revealing many of the same items as found at Vlasac (see above) and a similar settlement structure [1]. The site has been dated to the Lepenski Vir culture, and the most recent AMS dating suggests that the site was most heavily in use between ~6300 and 5700 cal BC (Lepenski Vir I-II & III) [4]. The Transitional phase in the Danube Gorges region is best represented at Lepenski Vir (I-II) (~6240-5845 calBC), where ~75 dwellings characteristic of the site were unearthed: buildings with trapezoidal limestone floors dug into the slope of the terrace [4, 8-10]. These trapezoidal buildings have been AMS dated to ~6300/6200 to 5900 cal BC, after which point most were abandoned and may have been primarily used for burials [4]; skeletal remains at the site were typically found in association with the house floors [10-12].

**Early Neolithic**

### *Vedrovice (southern Moravia, Czech Republic)*

The early LBK cemetery of Vedrovice in southern Moravia in the Czech Republic is located 40 kms southwest of Brno [13]. The site complex has been of particular interest due to its likely position on the earliest periphery of the Neolithic dispersal of agriculture into Central Europe [14-17]. Vedrovice is the earliest known LBK cemetery and has yielded the oldest radiocarbon date for a Central European LBK cemetery (5480 BC with highest probability between ~5300-5100 cal BC) [18]. The site was excavated between 1975 and 1982, encompassing 4500 m^2^ that included the main settlement of Sídlištĕ (700 m^2^), its cemetery of Široká u lesa (excavated between 1975 and 1982), and the burial ground of U Vinklerovy cihelny [19-25]. The site has yielded 85 burials and a rich record of daily life via its large range of preserved material culture [19-21, 25-33].

The skeletal remains from Vedrovice have been extensively studied and were the subject of the Vedrovice Bioarchaeology Project between 2005-2007, with results published in 2008 [13-14, 18, 34-41]. These analyses and others have allowed for major insight to be gained into the culture, diet, social structure, and migration patterns of the LBK. Analyses of stable isotopes and burial customs in LBK communities demonstrate a consistent pattern of patrilocal migration, differential access to cultivatable land, typically along kinship lines, and economic specialization in livestock herding or crop farming [42-49].

### *Nitra Horné Krškany (western Slovakia)*

The early LBK cemetery of Nitra Horné Krškany in western Slovakia dates to the Early Neolithic (5370-4980 cal BC) [50]. Pavúk [51] first documented the grave rituals and material culture of the cemetery, and subsequent analyses have included demography and paleopathology [52], stable isotopes [46], ancient DNA [53], and dental metrics, non-metrics, and pathology [54]. As was the case with Vedrovice, grave assemblages recovered from Nitra were rich and complex, and exhibit status differences by age and sex [55].

###

### *Schwetzingen (Baden-Württemberg, Germany)*

Schwetzingen is one of the largest LBK cemeteries known, with 203 graves excavated from the bank of the Rhine river in southwest Germany [56-58]. The cemetery was used between ~5260-5010 cal BC [46], and is notable for the low percentage of graves which were provided with grave goods (~50%) [59]. Where present, grave goods included arrowheads, polished points of stone and bone, and jewelry such as armrings and pendants [59].

### *Stuttgart-Mühlhausen (Baden-Württemberg, Germany)*

Stuttgart-Mühlhausen is a settlement and cemetery site located 20 kms north of Stuttgart and 80kms from Schwetzingen. A total of 247 burials were excavated from the burial ground, Viesenhäuser Hof, with 177 thought to belong to the LBK culture [43]. The cemetery consists of two distinct grave areas: 84 graves were excavated from Area I in the south section between 1977-1982, dating to the middle-late LBK, while 93 graves were excavated from Area II in the north section of the site between 1991-1993, dating to the early LBK [43]. Individuals excavated from Area II are indicated in Supplementary Table 1, while the remainder utilized in the current study are from Area I. Interestingly, though there are many similarities in LBK social structure and diet, grave assemblages in the Moravian and Slovakian LBK cemeteries of Vedrovice and Nitra were particularly rich and complex relative to those of southwest Germany (Stuttgart-Mühlhausen and Schwetzingen) and of the Linear Pottery cultures east of the Danube (e.g., at Polgár-Ferenci-hát) [50, 60].

### *Polgár-Ferenci-hát M3-31 (Great Hungarian Plain, Hungary)*

East of the Danube, early farmers belonged to the Alföld Linear Pottery (ALP) group. The Alföld region, also known as the Great Hungarian Plain, covers 150,000 kms^2^ of the central Carpathian Basin, ranging as far south as the Vojvodina province in northern Serbia [60]. Polgár-Ferenci-hát M3-31 is located ~40-60 kilometers from Füzesabony, in Hungary, and was discovered during the construction of the M3-31 motorway. Approximately 41,000 m^2^ was excavated between 2001- 2002 [61-62]. Polgár-Ferenci-Hát M3-31 dates to 5293-5068 cal BC [63], and consists of two phases of ALP occupation (I and II-IV). In the later phase, a central portion surrounded by a circular ditch could be identified, in which portions of burnt houses, a thick layer of debris and refuse, and round storage pits were discovered, indicative of intensive settlement [63]. This cemetery produced the largest number of ALP burials in the Tisza region, with 113 graves excavated in addition to settlement features [60]. Aside from pottery, vessels, red ochre, and stone tools, some of the deeper, waterlogged layers of the graves produced the earliest preserved traces of wooden coffins in Hungary [60]. However, the majority of the graves did not contain grave goods (72%) [60]. Anthropological analyses were performed by Zsuzsanna Zoffman and are summarized in Whittle and colleagues [60], including demography, stature reconstruction, and incidence of dental disease, paleopathology, and trauma.

**Middle Neolithic**

### *Hrtkovci-Gomolava (Vojvodina, northern Serbia)*

The large, multi-layered tell site of Gomolava is located on the bank of the river Sava, 55 kms west of Belgrade near the village of Hrtkovci in Vojvodina, northern Serbia, on the southern edge of the Carpathian Basin [64]. Gomolava has been known since 1898, with preliminary excavation carried out in 1904 and 1908 and larger-scale systematic excavation from 1953-1957 and 1969 through 1985 [64-70]. The site itself covers ~13,800 m^2^ and documents fairly continuous habitation from the Middle Neolithic through Medieval times [64]. The Vinča culture spans ~5300-4500 cal BC [71-72]; the Vinča layers at Gomolava represent the only known cemetery of Late Vinča culture and were radiocarbon dated to ~4950-4600 cal BC [64]. Preliminary anthropological analyses of the Vinča skeletal remains were published by Schwidetzsky [73] and Živanović [74], the latter of which documented an average male stature of 165.5 cms, just slightly taller than the average stature identified in the current study (163.98 cms). The Vinča layers at Gomolava revealed many copper artifacts, such as copper beads, bracelets, and chisels [64].

**Eneolithic**

*Hoštice 1 za Hanou (Moravia, Czech Republic)*

The Bell Beaker burial site of Hoštice is named for the nearby central Moravian village of Hoštice za Hanou in the Czech Republic. The Bell Beaker group is first seen in Central Europe in ~2900 BC [75], and expanded rapidly across Europe from ~2500 to 2000 BC, from Portugal in the west to Hungary in the east, and moved into Central Europe along the Lower Rhine River [76], where it was established by at least 2400 cal BC [77]. The culture is named for its characteristic bell-shaped and richly decorated beakers. The greatest concentration and richness of Bell Beaker burials in Europe comes from Moravia in the Czech Republic, and it is from here that the only Eneolithic cemetery included in analyses comes, the Bell Beaker site of Hoštice in Moravia.

The site of Hoštice was discovered during the construction of the D1 highway in 2002, and dates to ~2600 to 2000 BC. It is one of the largest Bell Beaker burial sites thus far discovered, with the remains of 150 individuals discovered [78]. Sex-specific burial rites and grave goods were common among the Bell Beakers: men were buried on their left side with their head northwards, often with stone arrowheads, copper daggers, and archery leg-plates, while women were buried on their right side with their head southwards, often with needles, bodkins, bone pendants, and/or earrings. Because only *some* men were buried with the eponymous beakers, copper and flint daggers, and archery equipment such as leg plates, it is possible that a differentiated warrior class existed among Bell Beaker society [79]. Occasionally, typically-male assemblages can be found buried with skeletons in typically-female burial position [80-82].

**Early/Middle Bronze Age**

### *Brno-Tuřany (Moravia, Czech Republic)*

The dominant culture in Early Bronze Age Moravia and Bohemia (Czech Republic) belonged to the Únětice, stretching across western Slovakia, Austria and Bavaria [83-84]. The more complex social hierarchy of the Bronze Age was visible in Únětice settlement patterns: small, fortified and strategically positioned centers of economic, political, and/or religious power were interspersed with small farming villages [85]. Únětice culture is represented in the current study from the settlement and burial site of Brno-Tuřany in Moravia, Czech Republic. Archaeological and anthropological details of the site of Brno-Tuřany were published in 2008 [86], and the site dates to the Moravian Early Bronze Age (approximately 2300-1700 BC) [19]. Únětice cemeteries have very consistent material culture and burial style across a wide expanse of Central Europe [84], and archaeological finds from the site include bronze hair ornaments and pottery fragments typical of the Únětice culture [86].

### *Polgár-Kenderföld (Great Hungarian Plain, Hungary)*

The Middle Bronze Age tell cemetery site of Polgár-Kenderföld is one of four cemeteries of the Füzesabony culture found within a few kilometers of each other in the Polgár region of Hungary, and was first excavated between 1989 and 1993 [87]. No specific dating has yet been performed, but a preliminary site report was published in 2001 [87], in which the strict burial rituals and ceramic grave goods were attributed to the Füzesabony culture. Traces of wooden coffins were visible in 35%+ of the burials, and grave goods included amber beads and bronze objects. Though the distribution of grave goods suggests vertical social differentiation in the Füzesabony communities living along the Tisza River in Early and Middle Bronze Age Hungary, this social differentiation did not appear to be reflected in major differences in health between men and women [88].

### *Ostojićevo (Vojvodina, northern Serbia)*

The Maros culture developed in the southern part of the Great Hungarian Plain during the Early Bronze Age, likely from local Copper Age Bodrogkeresztúr and immigrant Baden populations [89]. It was a long-lived culture, spanning from ~2500-1500 BC [90]. Maros groups lived in small farming villages, where they grew predominantly emmer wheat and barley, and tended sheep, goat, cattle, pigs, and horses [91]. Socioeconomic development was high among the Maros: the Maros group is particularly known for its large inhumation cemeteries containing varied and numerous grave goods including gold or rare shells and beads [92]. Ostojićevo in northern Serbia is one of these large Maros necropoli, and dates to approximately 1600/1500 BC, at the transition from the Early to Middle Bronze Age in the Vojvodina region [92]. Ostojićevo is located 24 kms northwest of Kikinda [93] and is one of the largest necropolises in Serbia, encompassing 3886 m^2^. The site was first discovered during the excavation of a dam in 1954, and was excavated between 1981 and 1995 by archaeologists with the National Museum of Kikinda, producing 77 Maros burials [93]. Grave goods were diverse and very clearly distributed according to social status and sex: daggers and axes were exclusively male high status items, while bone needles and bone and metal pins were exclusively female items, and headdresses were indicative of high status in both sexes [93].

**Iron Age**

### *Hrtkovci-Gomolava (Vojvodina, northern Serbia)*

In the Balkans south of the Sava and Danube rivers in northern Serbia, the transition from Late Bronze Age to Early Iron Age was marked by the emergence of the Bosut culture (~850 BC; Tasić, 2004). The Bosut culture was long-lived and spanned most of the Early Iron Age, from the 9th to 6th centuries BC [65], yet few skeletal remains are known from this time in Vojvodina [94]; thus, the Bosut layers at Gomolava in northern Serbia are particularly important. The Early Iron Age Bosut remains from Gomolava, site near Hrtkovci, in Vojvodina, northern Serbia [65] are dated by pottery to ~850-600/500BC. Collective burial pits were first discovered at Gomolava in 1971, in which the remains of both children and adults were placed simultaneously. There was no evidence of large-scale trauma or disease, and the inclusion of cereal grains, faunal remains, and grave goods placed on or near the remains suggests that burial rituals were followed [65]. Grave goods were diverse and included many bronze and iron bracelets, bronze beads, buttons, plaques, and rings, as well as bone, amber, and clay beads. Pottery vessels at the site indicate that the remains were buried in the earliest phase of Bosut culture, prior to the influx of Basarabi cultural elements from nearby Romania [65]. A gap in the habitation of Gomolava after its Early Iron Age phase suggests that the end of the Bosut culture coincided with the end of the occupation at Gomolava [95].

### *Brno-Maloměřice (Moravia, Czech Republic)*

The Middle Iron Age site of Brno-Maloměřice in the Czech Republic is the largest Celtic burial ground in Moravia [96]. The site dates to approximately 400-200 BC [97], from the time of the Celtic expansion into Central Europe [98]. Site and anthropological descriptions have been published by Poulík [99], Dacík [100], Čižmářová [97], and Trubačová [101]. The site was originally excavated in 1941 by Josef Poulík of the Moravian Museum, who uncovered 76 graves [102]. Burials at Brno-Maloměřice were by inhumation, and appear to have been indicated at the surface in some way, as they were arranged in regular groups or rows and never positioned on top of one another [102]. There is also evidence of enclosures or barriers at the site. Brno-Maloměřice has produced exceptional bronze decorated objects [102-103], including particularly elaborate metal fittings from a wooden jug considered to be one of the most complex and beautiful examples of Celtic art. The necropolis of Brno-Maloměřice demonstrates the high stratification of Celtic society: warriors, afforded high social standing, were buried with a much richer variety of grave goods and weapons than were non-warriors [102].

### *Tápiószele (Hungary)*

The Middle to Late Iron Age Scythian cemetery of Tápiószele in Hungary dates to 385 BC to 100 AD [104]. The site was excavated in 1938, 1941-1943, 1948, 1950-1953 and 1958 [105-106], producing 455 Scythian graves, the contents of many of which were destroyed in 1944-45 and 1956 [107]. Cemetery material was described in Blaskovich and Blaskovich [106] and Párducz [108], and anthropological investigations of Tápiószele were published by Bottyán [109], Ubelaker and Pap [104] and Fóthi and colleagues [107].

**Early Medieval Period**

### *Pottenbrunn (Lower Austria)*

In Austria, evidence of Slavic groups appears by the late 6th century AD [110]. Slavic farmers lived in small villages of wooden or clay huts, cultivating wheat and rye, and tending livestock [110-111]. The Early Medieval Slavonic site of Pottenbrunn in Lower Austria is dated by burial objects and costume details to ~800-850AD [112]. It was excavated from 1965 to 1974 [113], revealing a total of 172 graves containing 199 skeletons, 49 male, 51 female, and 99 children or adults of unidentified sex [110]. Grave goods included jewelry, tools, domestic animal bones, and egg shells.

**References**

1. Srejović D, Letica Z. 1978. Vlasac: A Mesolithic Settlement in the Iron Gates Region: Volume 1- Archaeology. Belgrade: Serbian Academy of Sciences and Arts Monographies Vol DXII, Department of Historical Sciences Vol. 5.

2. Borić D, French CAI, Stefanović S, Dimitrijević V, Cristiani E, Gurova M, Antonović D et al., 2014. Late Mesolithic lifeways and deathways at Vlasac (Serbia). J Field Archaeol 39:4-31.

3. Wallduck RJ. 2013. Post-mortem body manipulation in the Danube Gorges' Mesolithic-Neolithic: A taphonomic perspective. PhD dissertation, University of Cambridge.

4. Borić D, Dimitrijević V. 2007. When did the 'Neolithic package' reach Lepenski Vir? Radiometric and faunal evidence. Documenta Praehistorica 34:53-72.

5. Porčić M, Nikolić M. 2015. The Approximate Bayesian Computation approach to reconstructing population dynamics and size from settlement data: Demography of the Mesolithic-Neolithic transition at Lepenski Vir. Archaeol Anthropol Sci 1-18; DOI 10.1007/s12520-014-0223-2

6. Srejović D. 1969. Lepenski Vir - Nova praistorijska kultura u Podunavlju. Belgrade: Srpska književna zadruga.

7. Srejović D. 1972. Europe’s first monumental sculpture: new discoveries at Lepenski Vir. London: Stein and Day.

8. Borić D. 2002. The Lepenski Vir conundrum: reinterpretation of the Mesolithic and Neolithic sequences in the Danube Gorges. Antiquity 76:1026-1039

9. Borić D. 2011. Adaptations and Transformations of the Danube Gorges Foragers (c. 13.000–5500 BC): An overview. In: Krauß R, editor. Archaeolical and Anthropological Sciences Beginnings—New research in the appearance of the Neolithic between Northwest Anatolia and the Carpathian Basin; Papers of the International Workshop 8th–9th April 2009, Istanbul. Leidorf, Rahden/Westf. pp 157–203.

10. Radovanović I. 2000. Houses and burials at Lepenski Vir. Eur J Archaeol 3:330–349

11. Borić D, Dimitrijević V. 2007. Absolute chronology and stratigraphy of Lepenski Vir. Starinar 9–55.

12. Borić D, Stefanović S. 2004. Birth and death: infant burials from Vlasac and Lepenski Vir. Antiquity 78:582–601

13. Dočkalova M. 2008. Anthropology of the Neolithic population from Vedrovice (Czech Republic). Anthropologie 46: 239-315.

14. Lukes A, Zvelebil M, Pettitt P. 2008. Biological and cultural identity of the first farmers: Introduction to the Vedrovice Bioarchaeology Project. Anthropologie 46:117-124.

15. Zvelebil M. 2000. The social context of the agricultural transition in Europe. In: Renfrew C, Boyle K, editors. Archaeogenetics: DNA and the population prehistory of Europe. Cambridge: McDonald Institute Monographs. pp 57–79.

16. Zvelebil M. 2004. Conclusion: The many origins of the LBK. In: Lukes A, Zvelebil M, editors. LBK Dialogues: Studies in the formation of the Linear Pottery Culture. BAR International Series 1304. Oxford: Archaeopress. pp 183–205.

17. Zvelebil M, Pettitt P. 2013. Biosocial archaeology of the Early Neolithic: Synthetic analyses of a human skeletal population from the LBK cemetery of Vedrovice, Czech Republic. J Anthropol Archaeol 32:313-329.

18. Pettitt P, Hedges R. 2008. The age of the Vedrovice cemetery: The AMS radiocarbon dating programme. Anthropologie 46:125-134.

19. Podborský V. 1993. Pravěké dějiny Moravy. Brno: Muzejní a Vlastivĕdná Společnost v Brně.

20. Podborský V. 2002. Dvě pohřebiště Neolitického lidu s lineární keramikou ve Vedrovicích na Moravě. Brno: Ústav archeologie a muzeologie filosofické fakulty Masarykovy Univerzity.

21. Humpolová A. 2001. Rondeloid číslo III lidu s Moravskou malovanou keramikou ve Vedrovicích. In: Podborský V, editor. 50 let archeologický ch vý zkumů Masarykovy univerzity na Znojemsku. Brno: Ústav archeologie a muzeologie filosofická fakulta Masarykovy univerzity. pp 157–166.

22. Humpolová A, Ondruš V. 1999. Vedrovice, okres Znojmo. In: Podborský V, editor. Pravěká sociokulturní architektura na Moravě. Brno: Ústav archeologie a muzeologie filosofická fakulta Masarykovy univerzity. pp 167–219.

23. Čižmář Z. 2002. Keramika z pohřebiště "Široká u Lesa". In: Podborský V, editor. Dvě pohřebiště neolitického lidu s lineární keramikou ve Vedrovicích na Moravě. Brno: Ústav Archeologie a Muzeologie Filozofické Fakulty Masarykovy Univerzity. pp 151–190.

24. Dočkalova M, Čižmář Z. 2008. Antropologie a pohřbívání v moravském neolitu. In: Čižmář Z, editor. Život a Smrt v Mladší Době Kamenné. Brno: Ústav archeologické památkové péče, pp 236–247.

25. Ondruš V. 2002. Dvě pohřebiště Lidu s Neolitickou lineární keramikou ve Vedrovicích. In: Podborský V, editor. Dvě pohřebiště Neolitického lidu s lineární keramikou ve Vedrovicích na Moravě. Brno: Ústav archeologie a muzeologie filosofická fakulta Masarykovy univerzity. pp 9–122.

26. Ondruš V. 1961–1974. Nálezové deníky z výzkumných sezón 1961–1974. Nepublikované rukopisy uloženy na Archeologickém oddělení Moravského zemského muzea v Brně (Logbooks of finds from the research seasons 1961–1974. Unpublished manuscripts deposited at the Archaeological department of the Moravian Museum in Brno).

27. Ondruš V. 1963. Sídliště s keramikou volutovou z Vedrovic. Časopis Matice moravské Brno 46:51-57

28. Ondruš V. 1965. Neolitické hliněné plastiky z Vedrovic, Časopis Matice moravské Brno 50:31-36.

29. Ondruš V. 1972. Dětské pohřby na neolitickém sídlišti ve Vedrovicích. Časopis Matice moravské Brno 57:27-36.

30. Crubézy E, Murail P, Bruzek J, Jelínek J, Ondruš V, Pavúk J, et al. 1997. Sample characterization of Danubian cemeteries in Central Europe: the examples of Vedrovice (Moravia) and Nitra-Horne Krskany (Slovakia). Le Néolithique Danubien est ses marges entre Rhin et Seine. Actes du 22e colloque interrégional sur le Néolithique. Cahiers de l'Association pour la Promotion de la Recherche Archéologique en Alsace. pp 9–15.

31. Berkovec T. 2003. Plastika na sídlišti kultury s lineární keramikou ve Vedrovicích (okr. Znojmo). Ročenka 2003, Olomouc: Archeologické centrum Olomouc. pp 46–62.

32. Berkovec T, Veselá B. 2003-2004. Pece na sídlišti kultury s lineární keramikou ve Vedrovicích. Sborník Prací Filosofické Fakulty Masarykovi Univerzity- Řada Archaeolgická M8 9:7-30.

33. Berkovec T, Dreslerová G, Nývlotova-Fišáková M, Švédová J. 2004. Bone industry of the Linear Pottery Culture (LBK) at Vedrovice, Moravia. In: Lukes A, Zvelebil M, editors. LBK dialogues: Studies in the formation of the Linear Pottery Culture. British Archaeological Reports International Series, 1304. Oxford: Archaeopress. pp 159-176.

34. Bramanti B. 2008. Ancient DNA: Genetic analysis of aDNA from sixteen skeletons of the Vedrovice collection. Anthropologie 46:153-160.

35. Jarošová I. 2008. Dietary inferences using buccal microwear analysis on the LBK population from Vedrovice, Czech Republic. Anthropologie 46:175-184.

36. Lillie M. 2008. Vedrovice: Demography and paleopathology in an early farming population. Anthropologie 46:135-152.

37. Nystrom P. 2008. Dental microwear signatures of an early LBK population from Vedrovice, Moravia, the Czech Republic. Anthropologie 46:161-173.

38. Richards MP, Montgomery J, Nehlich O, Grimes V. 2008. Isotopic analysis of humans and animals from Vedrovice. Anthropologie 46:185-194.

39. Smrčka V, Mihaljevic M, Sebek O, Humpolova A, Berkovec T, Dočkalová M. 2008. Trace-elemental analysis of skeletal remains of humans and animals at the Neolithic settlement in Vedrovice (Czech Republic). Anthropologie 46:219-226.

40. Smrčka V, Erban V, Hlozek M, Gregerová M, Dočkalová M. 2008. Reconstruction of mobility: Comparison between the analysis of Sr isotopes in a set of Neolithic skeletons from the Vedrovice cemetery, and the petrographical analysis of pottery in graves. Anthropologie 46:233-238.

41. Zvelebil M, Pettitt P. 2008. Human condition, life, and death at an early Neolithic settlement: Bioarchaeological analyses of the Vedrovice cemetery and their biosocial implications for the spread of agriculture in Central Europe. Anthropologie 46:195-218.

42. Price D, Bentley RA, Lüning J, Gronenborn D, Wahl J. 2001. Prehistoric human migration in the Linearbandkeramik of Central Europe. Antiquity 75:593–603.

43. Price TD, Wahl J, Knipper C, Burger-Heinrich E, Kurz G, Bentley RA. 2003. Das bandkeramische Gräberfeld von Stuttgart-Mühlhausen: Neue Untersuchungsergebnisse zum Migrationsverhalten im frühen Neolithikum. In: Funda, DT, editor. Fundberichte aus Baden-Württemberg. Stuttgart: Kommissionsverlag Konrad Theiss Verlag. pp 23-58.

44. Bentley RA. 2012. Mobility and the diversity of early Neolithic lives: Isotopic evidence from skeletons. J Anthropol Archaeol 32:303-312.

45. Bentley RA, Price TD, Wahl J, Atkinson TC. 2008. Isotopic signatures and hereditary traits: Snapshot of a Neolithic community in Germany. Antiquity 82:290–304.

46. Bentley RA, Bickle P, Fibiger L, Nowell GM, Dale CW, Hedges REM, et al. 2012. Community differentiation and kinship among Europe’s first farmers. P Natl Acad Sci 109:9326-9330.

47. Knipper C, Price TD. 2010. Individuelle Mobilität in der Linearbandkeramik: Strontiumisotopenanalysen vom Gräberfeld Stuttgart-Mühlhausen „Viesenhäuser Hof“. In: Krenn-Leeb A, Beier HJ, Classen E, Falkenstein F, Schwenzer S, editors. Mobilität, Migration und Kommunikation in Europa während des Neolithikums und der Bronzezeit. Beiträge der Sitzungen der Arbeitsgemeinschaften Neolithikum und Bronzezeit während der Jahrestagung des West- und Süddeutschen Verbandes für Altertumsforschung. Mosen: Beier und Beran. pp 53-63.

48. Knipper C. 2009. Mobility in a sedentary society: Insights from isotope analysis of LBK human and animal teeth. In: Hofmann D, Bickle P, editors. Creating communities: New advances in Central European Neolithic research. Oxford and Oakville: Oxbow Books. pp 142-158.

49. Bogaard A, Krause R, Strien HC. 2011. Towards a social geography of cultivation and plant use in an early farming community: Vaihingen an der Enz, south-west Germany. Antiquity 85:395-416.

50. Whittle A, Bentley RA, Bickle P, Dočkalová M, Fibiger L, Hamilton J, et al. 2013. Moravia and western Slovakia. In: Bickle P, Whittle A, editors. The first farmers of Central Europe: Diversity in LBK lifeways. Oxbow Books: Oxford. pp 101-158.

51. Pavúk J. 1972. Neolithisches Gräberfeld in Nitra. Slovenská Archeologia 20:5-105.

52. Crubézy E, Goulet J, Bruzek J, Jelinek J, Rougé D, Ludes B. 2002. Epidemiology of osteoarthritis and enthesopathies in a European population dating back 7700 years. Joint Bone Spine 69:580-588.

53. Haak W, Forster P, Bramanti B, Matsumura S, Brandt G, Tänzer M, Villems R, Renfrew C, Gronenborn D, Alt KW. 2005. Ancient DNA from the first European farmers in 7500-year-old Neolithic sites. Science 310:1016-1018.

54. Frayer DW. 2004. The dental remains from Krškany (Slovakia) and Vedrovice (Czech Republic). Anthropologie 42:71-103.

55. Milisauskas S. 2002. Early Neolithic: The first farmers in Europe, 7000-5500/5000 BC. In: Milisauskas S, editor. European prehistory: A survey. New York: Kluwer Academic. pp 143-192.

56. Nieszery N. 1995. Linearbandkeramische Gräberfelder in Bayern. Internationale Archäologie 16.

57. Gerling C. 2009. Schwetzingen, ein 'reguläres' Gräberfeld der jüngeren Linearbandkeramik. In: Zeeb-Lanz A, editor. Krisen-Kulturwandel-Kontinuitäten. Zum Ende der Bandkeramik in Mitteleuropa. Beiträge der internationalen Tagung in Herxheim bei Landau (Pfalz) vom 14-17.06.2007. Rahden: Marie Leidorf. pp 103-110.

58. Gerling C, Francken M. 2007. Das linearbankeramische Gräberfeld von Schwetzingen. Archäologische Informationen 30:43-50.

59. Bentley RA, Bickle P, Francken M, Gerling C, Hamilton J, Hedges R, Stephan E, Wahl J, Whittle A. 2013. Baden-Württemberg. In: Bickle P and Whittle A, editors. The first farmers of Central Europe: Diversity in LBK lifeways. pp 251-288.

60. Whittle A, Anders A, Bentley RA, Bickle P, Cramp L, Domboróczki L, Fibiger L, Hamilton J, Hedges R, Kalicz N, Kovács ZE, Marton T, Oross K, Pap I, Raczky P. 2013. Hungary. In: Bickle P, Whittle A, editors. The first farmers of Central Europe: Diversity in LBK lifeways. Oxford: Oxbow Books. pp 49-100.

61. Raczky P. 2004. Polgár, Ferenci-hát. In: Kisfaludi J, editor. Régészeti Kutatások Magyarországon 2002 (Archaeological Investigations in Hungary 2002), Budapest. pp 257-258.

62. Raczky P. 2007. Az autópálya-régészet helyzete Magyarországon: Módszerek és tapasztalatok az 1990 és 2007 közötti munkálatok alapján. [English: The state of “motorway archaeology” in Hungary: Methods and experience based on the work carried out between 1990 and 2007] Archaeologiai Értesítő 132:5-36.

63. Raczky P, Anders A. 2009. Settlement history of the Middle Neolithic in the Polgár micro-region (the development of the Aföld Linearband Pottery in the upper Tisza region, Hungary. In: Kozlowski JK, editor. Interactions between different models of Neolithization north of the central European agro-ecological barrier. Kraków: Polska Akademiai Umiejętności. pp 31-50.

64. Borić D. 2009. Absolute dating of metallurgical innovations in the Vinča culture of the Balkans. In: Kienlin TL, Roberts BW, editors. Metals and Societies: Studies in honour of Barbara S. Ottaway. Bonn: Habelt. pp 191-245.

65. Tasić N. 1972. An Early Iron Age collective tomb at Gomolava. Archaologia Iugoslavica 13:27-37.

66. Brukner B. 1965. Neolithic and early Eneolithic layers at Gomolava. Rad Vojvodanski Muzeiu 14:137-175.

67. Brukner B. 1971. Gomolava à Hrtkovci, site préhistorique à plusieurs couches. In: Novak G, editor. Epoque Préhistorique et Protohistorique en Yougoslavie. Recherches et Résultats. Beograd: Societé archeologique de Yougoslavie. pp 175-176.

68. Brukner B. 1973. Die Stratigraphie von Gomolava. Ein Vorschlag zur Bestimmung der relativen Chronologie an dem Ubergang vom Endneolithikum zurn Aneolithikum. Actes du VZZe Congres International des Sciences Prehistoriques et Protohistoriques 2:317-324.

69. Jovanović B. 1965. General stratigraphy. Gomolava-excavation from 1965-1966. Rad Vojvodanski Muzeia 14:113-l15.

70. Jovanović B. 1971. Stratigraphy of Gomolava in excavation 1967-71. Rad Vojvodanski Muzeia 20:95-102.

71. Breunig P. 1987. ^14^C Chronologie des vorderasiatischen, südost- und mitteleuropäischen Neolithikums [^14^C chronology of the Preasiatic, South-East and Middle-European Neolithic]. Vol. 13. Köln: Kommission bei Böhlau.

72. Gläser R. 1996. Zur absoluten Datierung der Vinča-Kultur anhand von 14C-Daten. In: Draşovean F, editor. The Vinča culture: Its Role and cultural connections. Timişoara: The Museum of Banat. pp 175–212.

73. Schwidetzsky I. 1971-72. Menschliche skelettreste von Vinca. Glasnik Antropoloskog Drustva Jugoslavije 8-9:101-12.

74. Živanović S. 1977. Vinča skeletons studied in situ at the Gomolava site, Yugoslavia. Curr Anthropol 18:533-534.

75. Forenbaher S. 1993. Radiocarbon dates and absolute chronology of the Central European Early Bronze Age. Antiquity 67:218-256.

76. Grupe G, Price TD, Schröter P, Söllner F, Johnson CM, Beard BL. 1997. Mobility of Bell Beaker people revealed by strontium isotope ratios of tooth and bone: A study of southern Bavarian skeletal remains. Applied Geochemistry 12:517-525.

77. Lee EJ, Makarewicz C, Renneberg R, Harder M, Krause-Kyora B, Müller S, Ostritz S, Fehren-Schmitz L, Schreiber S, Müller J, von Wurmb-Schwark N, Nebel A. 2012. Emerging genetic patterns of the European Neolithic: Perspectives from a late Neolithic Bell Beaker burial site in Germany. AJPA 148:571-579.

78. Vaňharová M, Drozdová E. 2008. Sex determination of skeletal remains of 4000 year old children and juveniles from Hoštice 1 za Hanou (Czech Republic) by ancient DNA analysis. Anthropological Review 71:63-70.

79. Sarauw T. 2007. Male symbols or warrior identities? The ‘archery burials’ of the Danish Bell Beaker culture. J Anthropol Archaeol 26:65–87.

80. Dvořák P, Hájek L. 1990. Die Gräberfelder der Glockenbecherkultur bei Šlapanice (Bez. Brnovenkov). Brno: Katalog der Funde, MAQ.

81. Turek J, Foster PJ. 2000. Výzkum polykulturního naleziště v Tišicích (okr. Mělník). Archeologické výzkumy v Čechách 1999, Zprávy České archeologické společnosti, Supplement 42: 6-7.

82. Turek J. 2006. Beaker barrows and the houses of dead. In: Šmejda L, editor. Archaeology of Burial Mounds. Plzeň Czech Republic: University of West Bohemia. pp. 170-179.

83. Coles JM, Harding AF. 1979. The Bronze Age in Europe: An introduction to the prehistory of Europe c. 2000-700BC. London: Methuen.

84. Harding A. 2000. European societies in the Bronze Age. Cambridge: Cambridge University Press.

85. Greenfield HJ. 2001. Únětice culture. In: Peregrine P, Ember M, editors. Encyclopaedia of Prehistory: Vol. 4, Europe, for the Human Area Relations Files. New York: Kluwer Academic/Plenum Publishers. pp 134-137.

86. Kala J, Konášová K, Smrčka V. 2008. Pohřby ze zásobních jam na okraji únětického pohřebiště v Brně-Tuřanech. In: Stuchlík S, Ghosh V, Janak V, editors. Acta Archaeologica Opaviensia. Opava: Ústav historických věd FPF Slezské Univerzity v Opavě. pp 61-78.

87. Dani J, Máthé M, Szabö G. 2001. Ausgrabungen in der bronzezeitlichen Tell-Siedlung und im Gräberfeld von Polgár-Kenderföld (Vorbericht über die Freilegung des mittelbronzezeitlichen Gräberfeldes von Polgár-Kenderföld, Majoros-Tanya). In: Kacsó C, editor. Bronzezeitliche Kulturerscheinungen im Karpatischen Raum. Die Beziehungen zu den benachbarten Gebieten. Ehrensymphosium für Alexandru Vulpe zum 70. Geburtstag, Baia Mare Oktober 10-13, 2001. pp 93-118.

88. Ubelaker DH, Pap I. 1996. Health profiles of a Bronze Age population from northeastern Hungary. Annls Hist Nat Mus Nat Hung 88:271-296.

89. Zoffmann ZK. 2000. Anthropological sketch of the prehistoric population of the Carpathian Basin. Acta Biologica Szegediensis 44:75-79.

90. O'Shea J. 1992. A radiocarbon-based chronology for the Maros group of south-east Hungary. Antiquity 66:97-102.

91. Greenfield HJ. 2001. The Maros culture: a regional subtradition during the Early Bronze Age of Central Europe. In: Peregrine P, Ember M, editors. Encyclopaedia of Prehistory: Vol. 4, Europe, for the Human Area Relations Files. New York: Kluwer Academic/Plenum Publishers. pp 150-153.

92. Tasić N. 2003. Historical picture of development of Bronze Age cultures in Vojvodina. Starinar LIII-LIV:23-34.

93. Milašinović L. 2009. Review on possibility of social structure reconstruction on the Maros culture finds from the necropolis at Ostojićevo. English summary. Rad Muzeja Voyvodina 51:65-70.

94. Zoffmann ZK. 2011. Antropološka analiza skeleta iz ranog gvozdenog sa arheološkog lokaliteta Vranj-Hrtkovci. Rad Muzeja Vojvodine 53:93-96.

95. Tasić N. 2005. Bronze and Iron Age sites in Srem and the stratigraphy of Gomolava. Balcanica 36:7-16.

96. Dreslerová G. 2005. Vyhodnocení zvířecích kostí. Brno: Ústav archeologické památkové péče Brno. Pravěk Supplementum 14.

97. Čižmářová J. 2005. Keltské pohřebiště v Brně-Maloměřicích / Das keltische Gräberfeld in Brno-Maloměřice. Brno: Pravěk supplementum 14. 226 p.

98. Barford P. 1991. Celts in Central Europe and beyond. Archaeologia Polona 29:79-98.

99. Poulík J. 1942. Das keltische Gräberfeld von Brünn-Malmeritz. Zeitschrift des Mährischen Landesmuseums 2:49-86.

100. Dacík T. 1983. Příspěvek k antropologii Keltů na Moravě. Archeologické rozhledy 35:496-509.

101. Trubačová T. 2005. Rozbor antropologického materiálu. In: Čižmářová J, editor. Keltské pohřebiště v Brně-Maloměřicích / Das keltische Gräberfeld in Brno-Maloměřice. Brno: Pravěk supplementum 14. pp 27-40.

102. Čižmářová J. 2006. Les nécropoles celtiques de la Moravie. In: Dossiers Archeologie et sciences des origines No. 313:26-33.

103. Kruta V. 2007. La cruche celte de Brno. Chef-d’oeuvre de l’art. Dijon: Miroir de l’Univers.

104. Ubelaker DH, Pap I. 1998. Skeletal evidence for health and disease in the Iron Age of Northeastern Hungary. Int J Osteoarchaeol 8:231-251.

105. Bottyán Á. 1955. Szkíták a magyar Alföldön. Régészeti Füzetek 1. Budapest: Magyar Nemzeti Múzeum.

106. Blaskovich J, Blaskovich G. 1958. Tápiószele. Arch Ert 85:202.

107. Fóthi E, Zsolt B, Sándor E. 2006. A Tápiószelei szkíta kori temető embertani vizsgálata. In: Móró G, editor. Az aranyszarvas nyomában: A Blaskovich fiverek és a magyar régészet kapcsolata. Tápiószele: Blaskovich Múzeum Baráti Köre. pp 65-93.

108. Párducz M. 1966. The Scythian Age Cemetery at Tápiószele. Acta Archaeol 18:39-82.

109. Bottyán O. 1943. Szkítakori temetôk embertani vizsgálata. Acta Scientiarium Mathematicarum et Naturalium (Kolozsvár) 15:1-63.

110. Herold M. 2008. Sex differences in mortality in Lower Austria and Vienna in the Early Medieval Period: An investigation and evaluation of possible contributing factors. Universität Wien: PhD Dissertation.

111. Postan MM, 1966. The agrarian life of the Middle Ages. Cambridge: Cambridge University Press.

112. Friesinger, H. 1972. Frühmittelalterliche Körpergräber aus Pottenbrunn, Stadtgemeinde St. Pölten, NÖ. Archaeologia Austriaca 51:113-189.

113. Fabrizii-Reuer S, Reuer E. 2001. Das frühmittelalterliche Gräberfeld von Pottenbrunn, Niederösterreich: Anthropologische Auswertung. Mitteilungen der Prähistorischen Kommission der. Vienna: Verlag der Österreichischen Akademie der Wissenschaften.
