# Supplementary material for: Early Life Conditions and Physiological Stress following the Transition to Farming in Central/Southeast Europe: Skeletal Growth Impairment and 6000 Years of Gradual Recovery
Source: PLoS One. 2016 Feb 4;11(2):e0148468. doi: 10.1371/journal.pone.0148468 (PMC4742066; doi:10.1371/journal.pone.0148468)
Supplement: S2 Table — (DOCX) [file pone.0148468.s003.docx]

**Supplementary Table 2. Summary statistics for body size variables by sex and site**

| Region | Time Period | Site | Stature | | | Body Mass | | | Brachial Index | | | Crural Index | | |
| --- | --- | --- | --- | --- | --- | --- | --- | --- | --- | --- | --- | --- | --- | --- |
|  |  |  | N | Mean | SD | N | Mean | SD | N | Mean | SD | N | Mean | SD |
| **MALES** |  |  |  |  |  |  |  |  |  |  |  |  |  |  |
| *Vojvodina* | Mesolithic | Vlasac | 12 | 173.29 | 4.51 | 8 | 70.90 | 6.84 | 7 | 78.29 | 7.69 | 4 | 85.33 | 1.11 |
|  | Mesolithic | Lepenski Vir | 11 | 168.81 | 8.27 | 8 | 70.05 | 5.74 | 1 | 84.59 | - | 3 | 81.31 | 2.49 |
|  | Neolithic | Gomolava | 14 | 163.98 | 5.03 | 12 | 62.04 | 5.75 | 12 | 76.85 | 1.53 | 10 | 81.95 | 2.86 |
|  | Bronze Age | Ostojićevo | 28 | 163.96 | 7.38 | 26 | 66.01 | 10.08 | 18 | 76.71 | 1.80 | 22 | 82.80 | 1.84 |
|  | Iron Age | Gomolava | 6 | 153.36 | 4.09 | 7 | 56.29 | 7.58 | 4 | 75.15 | 2.98 | 4 | 86.00 | 3.57 |
| *Moravia/* |  |  |  |  |  |  |  |  |  |  |  |  |  |  |
| *Lower Austria* | Neolithic | Vedrovice | 10 | 162.11 | 4.08 | 10 | 60.69 | 5.97 | 8 | 77.75 | 2.30 | 10 | 81.70 | 2.08 |
|  | Eneolithic | Hoštice | 8 | 168.86 | 2.72 | 6 | 66.75 | 4.87 | 5 | 77.46 | 1.52 | 7 | 84.13 | 2.08 |
|  | Bronze Age | Brno-Tuřany | 9 | 164.16 | 3.31 | 8 | 66.62 | 2.59 | 8 | 78.58 | 2.15 | 9 | 85.53 | 1.71 |
|  | Iron Age | Brno-Maloměřice | 10 | 166.48 | 5.69 | 13 | 63.12 | 5.71 | 2 | 77.85 | 4.95 | 10 | 84.21 | 2.18 |
|  | Medieval | Pottenbrunn | 17 | 166.61 | 7.23 | 17 | 66.38 | 11.94 | 15 | 75.89 | 1.12 | 17 | 83.72 | 1.90 |
| *Hungary/* |  |  |  |  |  |  |  |  |  |  |  |  |  |  |
| *Slovakia* | Neolithic | Polgár-Ferenci-hát | 6 | 164.05 | 4.22 | 8 | 65.71 | 5.47 | 6 | 74.67 | 2.03 | 6 | 82.74 | 1.21 |
|  | Neolithic | Nitra | 10 | 164.40 | 2.97 | 11 | 65.07 | 7.74 | 10 | 75.80 | 1.34 | 9 | 83.33 | 2.49 |
|  | Bronze Age | Polgár Kenderföld | 15 | 163.80 | 5.01 | 15 | 62.26 | 6.06 | 11 | 76.86 | 3.38 | 11 | 85.81 | 2.53 |
|  | Iron Age | Tápiószele | 10 | 166.52 | 3.55 | 11 | 65.53 | 5.83 | 2 | 76.56 | 0.67 | 5 | 82.97 | 1.23 |
|  |  |  |  |  |  |  |  |  |  |  |  |  |  |  |
| *Baden-*  *Württemberg* | Neolithic | Schwetzingen | 13 | 162.48 | 5.65 | 14 | 60.66 | 4.65 | 4 | 78.37 | 1.00 | 10 | 82.97 | 2.13 |
|  | Neolithic | Stuttgart-Mühlhausen | 22 | 163.92 | 7.49 | 25 | 60.40 | 7.68 | 17 | 77.19 | 1.93 | 17 | 83.15 | 2.09 |
|  |  |  |  |  |  |  |  |  |  |  |  |  |  |  |
| **FEMALES** |  |  |  |  |  |  |  |  |  |  |  |  |  |  |
| *Vojvodina* | Mesolithic | Vlasac | 13 | 161.55 | 4.52 | 2 | 60.21 | 1.74 | 5 | 78.30 | 3.31 | 4 | 85.11 | 2.73 |
|  | Mesolithic | Lepenski Vir | 13 | 154.89 | 4.56 | 8 | 53.80 | 2.53 | - | - | - | 5 | 80.27 | 4.64 |
|  | Bronze Age | Ostojićevo | 27 | 151.87 | 4.73 | 27 | 54.17 | 4.35 | 20 | 75.02 | 1.37 | 22 | 83.38 | 1.26 |
|  | Iron Age | Gomolava | 13 | 152.59 | 5.56 | 15 | 52.65 | 4.09 | 6 | 74.32 | 3.03 | 9 | 84.11 | 2.61 |
| *Moravia/* |  |  |  |  |  |  |  |  |  |  |  |  |  |  |
| *Lower Austria* | Neolithic | Vedrovice | 12 | 152.05 | 5.76 | 12 | 50.93 | 4.18 | 9 | 74.95 | 1.84 | 9 | 82.13 | 1.50 |
|  | Copper Age | Hoštice | 2 | 152.24 | 0.38 | 3 | 53.82 | 5.70 | 1 | 76.98 | - | 1 | 85.79 | - |
|  | Bronze Age | Brno-Tuřany | 7 | 159.96 | 2.97 | 5 | 58.88 | 2.62 | 4 | 78.34 | 1.80 | 2 | 85.08 | 1.27 |
|  | Iron Age | Brno-Maloměřice | 4 | 153.50 | 4.73 | 4 | 56.87 | 1.41 | 2 | 73.21 | 1.55 | 4 | 84.09 | 1.59 |
|  | Medieval | Pottenbrunn | 23 | 158.52 | 4.31 | 21 | 55.60 | 6.99 | 11 | 75.01 | 1.68 | 21 | 84.32 | 1.29 |
| *Hungary/* |  |  |  |  |  |  |  |  |  |  |  |  |  |  |
| *Slovakia* | Neolithic | Polgár-Ferenci-hát | 2 | 150.15 | 1.61 | 2 | 53.22 | 2.18 | 1 | 73.63 | - | 2 | 79.41 | 2.48 |
|  | Neolithic | Nitra | 6 | 154.41 | 0.74 | 10 | 54.13 | 5.53 | 6 | 74.92 | 1.72 | 7 | 82.78 | 2.04 |
|  | Bronze Age | Polgár Kenderföld | 6 | 159.05 | 4.70 | 6 | 55.22 | 4.15 | 4 | 77.03 | 5.36 | 6 | 84.45 | 2.19 |
|  | Iron Age | Tápiószele | 14 | 153.81 | 4.07 | 14 | 56.68 | 2.35 | 8 | 76.59 | 1.81 | 6 | 84.52 | 1.30 |
|  |  |  |  |  |  |  |  |  |  |  |  |  |  |  |
| *Baden-*  *Württemberg* | Neolithic | Schwetzingen | 13 | 152.35 | 4.29 | 11 | 52.61 | 2.34 | 4 | 75.57 | 2.07 | 5 | 82.79 | 3.23 |
|  | Neolithic | Stuttgart-Mühlhausen | 14 | 151.40 | 7.24 | 14 | 50.95 | 5.06 | 11 | 75.28 | 1.63 | 11 | 82.24 | 1.77 |

N= # of individuals; SD= standard deviation
